# Dissecting Bread Wheat Heterosis through the Integration of Agronomic and Physiological Traits

**DOI:** 10.3390/biology10090907

**Published:** 2021-09-13

**Authors:** Kevin Gimenez, Pierre Blanc, Odile Argillier, Jean-Baptiste Pierre, Jacques Le Gouis, Etienne Paux

**Affiliations:** 1 Université Clermont Auvergne, INRAE, Genetics, Diversity and Ecophysiology of Cereals, 63000 Clermont-Ferrand, France; kevin.gimenez@inrae.fr (K.G.); jacques.le-gouis@inrae.fr (J.L.G.); 2 Syngenta France SAS, 28000 Chartres, France; pierre.blanc@syngenta.com (P.B.); odile.argillier@syngenta.com (O.A.); jean-baptiste.pierre@syngenta.com (J.-B.P.)

**Keywords:** hybrids, wheat, heterosis, yield factor, grain protein deviation, grain filling, senescence

## Abstract

**Simple Summary:**

To meet the challenge of feeding almost 10 billion people by 2050, wheat yield has to double by 2050. However, over the past 20 years, yield increase has slowed down and even stagnated in the main producing countries. Similar to what has been observed in maize, hybrids have been suggested as a solution to overcome yield stagnation in wheat. However, wheat heterosis, i.e., the fact that a progeny surpasses the performances of its parents, is still limited and poorly understood. To better characterize this phenomenon, we developed and phenotyped for physiological and agronomic traits 91 hybrids and their nineteen female and sixteen male parents. We showed that hybrids had a longer grain filling phase that led to bigger grains and an increased thousand kernel weight. This resulted in a better yield for 86% of hybrids compared to the average yield of their parents. In addition, hybrids appeared to be less affected by the negative correlation between protein content and yield compared to pure lines. These results shed light on the physiological bases underlying yield heterosis in wheat, paving new ways to breed for better wheat hybrids that can help to meet agriculture’s challenges.

**Abstract:**

To meet the challenge of feeding almost 10 billion people by 2050, wheat yield has to double by 2050. However, over the past 20 years, yield increase has slowed down and even stagnated in the main producing countries. Following the example of maize, hybrids have been suggested as a solution to overcome yield stagnation in wheat. However, wheat heterosis is still limited and poorly understood. Gaining a better understanding of hybrid vigor holds the key to breed for better varieties. To this aim, we have developed and phenotyped for physiological and agronomic traits an incomplete factorial design consisting of 91 hybrids and their nineteen female and sixteen male parents. Monitoring the plant development with normalized difference vegetation index revealed that 89% of the hybrids including the five higher yielding hybrids had a longer grain filling phase with a delayed senescence that results in larger grain size. This average increase of 7.7% in thousand kernel weight translated to a positive mid-parent heterosis for grain yield for 86% of hybrids. In addition, hybrids displayed a positive grain protein deviation leading to a +4.7% heterosis in protein yield. These results shed light on the physiological bases underlying yield heterosis in wheat, paving new ways to breed for better wheat hybrids.

## 1. Introduction

With 216 million hectares and an annual production of 765 million tons, bread wheat (*Triticum aestivum* L.) is one of the most important crops worldwide and the staple food for one third of the world population [1]. It is also a major renewable resource for feed and industrial raw materials. Today’s agriculture has to face an unprecedented challenge: to keep pace with the human demand in an environmentally and socially sustainable manner [2]. To feed a world population that is expected to reach 9.7 billion by 2050 [3], wheat yield should increase by 2.4% per year over the next 30 years [4]. This goal would be achievable under the assumption of favorable growing conditions but is unlikely under climate change that affects not only the average yield but also its stability [5,6,7]. Wheat yields have been multiplied by 2.5 between 1960 and 1995 as a result of genetic progress as well as the development of cultivation techniques that have been applied to crops since the “Green Revolution” [5,8,9]. However, yield increase has slowed down to an annual rate of approximatively 1% per year over the past 20 years, and even stagnating in the main producing countries such as Europe [10]. Indeed, with its high yielding wheat production, the EU27 is the first world wheat producer and its production contributes significantly (22%) to the world supply. France alone ranks fifth in the world and first in the EU for both production and export. However, like in many other countries, French annual yield has been stagnating since the end of the 1990s and the genetic progress was only able to compensate for more unfavorable growing conditions [5,11]. Thus, there is an urgent need to accelerate genetic progress for yield potential as well as to improve tolerance to biotic and abiotic stresses that are expected to increase in frequency and intensity as a consequence of climate change [12].

Yield increase slow-down has also been observed in other crops such as rice and soybean [4]. By contrast, maize is doing better, yet not sufficiently, with an annual rate of +1.6% since the beginning of the 21st century. This current success can be at least partially attributed to the development of hybrid varieties since the 1930s that led to a 5.5-fold yield increase over 75 years [13]. Even though this annual yield gain results from several complementary factors including agronomic practices and development of new varieties, the genetic gain for maize was the main factor for +100 kg/ha/year [14]. By contrast, average wheat genetic gain in Europe is about +40 kg/ha/year [15]. Consequently, the use of hybrid varieties appears as a possible way to boost yield increase in wheat. A study performed on wheat commercial breeding programs during 21 years in North America reported a genetic gain for hybrids of +1.55% per year compared to 0.84% for pure lines [16]. However, despite having been a matter of interest since the 1970s, the deployment of hybrid wheat has long been hampered by two interconnected factors. The first factor is that hybrid wheat production has long relied on the use of chemical hybridizing agents (CHA), that are difficult to use, and also a low rate of cross pollination, both difficulties resulting in high cost for seed production [17]. This leads to the second factor which is the yield heterosis needed to compensate additional cost for hybrid seed production. In the 1980s and 1990s, a 10% yield heterosis was often observed for some combinations of hybrid wheat in experimental trials [18,19] and up to 13.5% for the best hybrids after breeding [20]. However, very few hybrids overpassed the +13% threshold estimated for profitability [21] and the interest decreased and few acres were grown with hybrid wheat varieties [17]. During the 2010s, the advent of the wheat genome sequencing projects allowed for a better knowledge of the genes involved in the hybrid production (cytoplasmic male sterility and fertility restauration) leading to a new generation of hybrids with high heterosis [22,23,24,25,26]. Additional interest was also observed in hybrids that showed a better grain protein deviation and a higher yield stability [23,27].

Heterosis for grain yield has mainly been associated with thousand kernel weight [28,29], suggesting that hybrids and pure lines may differ for the dynamic of canopy senescence. Normalized Difference Vegetation Index (NDVI) has been proposed as a way to rapidly characterize senescence kinetics on a large number of genotypes [30,31]. Measured on pure lines, NDVI has highlighted the importance of specific stages in yield determination [30,31,32,33]. Integrative approaches using NDVI measurements revealed particularly that stay green traits resulting in a delayed senescence could be linked to increased yield [34,35,36,37].

The objective of our study was to (i) evaluate heterosis for agronomic and physiological traits and (ii) assess the potential of physiological traits estimated from NDVI to explain heterosis. Our study is based on an incomplete factorial design including 19 females, 16 males, and 92 F1 combinations, which were grown at three locations in northern France.

## 2. Materials and Methods

### 2.1. Plant Material and Genotyping

A set of 136 wheat varieties were selected within the genetic pool from Syngenta breeding company comprising 43 lines carrying fertility restorer (Rf) genes, hereafter referred to as ‘males’, and 93 lines carrying a cytoplasmic male sterility (CMS) derived from *Triticum timopheevii*, referred to as ‘females’ [38]. Genotyping was performed by Trait Genetics (Gatersleben, Germany) using the Illumina^®^ iSelect 15K wheat SNP array [39]. Heterozygous data were considered as missing data. Monomorphic markers and markers with more than 20% missing data were removed from the analysis. No minor allele frequency filtering was applied. SNPs were pruned for linkage disequilibrium (r^2^ = 1) using the plink software [40]. Eventually, a subset of 2966 SNPs was selected to describe the diversity within the genetic panel.

Pearson coefficient correlations were calculated with the R *cor* function for each male x female combination (use = “pairwise.complete.obs”) [41]. Dissimilarity matrices were built using the “as.dist (1-correlation)” function. Hierarchical clustering was performed using the “hclust” function (method = “average”) and genetic groups were identified using function “cutree”. Dendrogram representing the genetic distances between lines of the panel were produced using function “dendro_data_k” of package ggdendro [42]. Principal Component Analyses (PCAs) were conducted with the centered and scaled genotype matrix using package FactoMineR [43]. Individuals were projected onto the two first axes using the ggplot2 package [44].

The 35 parents selected from the genetic panel were genotyped with an improved Axiom array based on the TaBW280K SNP chip [45] and composed of 410K SNP markers which was conducted on the Affymetrix GeneTitan system according to the procedure described by Affymetrix (Axiom^®^ 2.0 Assay Manual Workflow User Guide Rev3, Santa Clara, CA, USA). Allele calling was carried out using a modified version of the Affymetrix proprietary software packages named Affymetrix Power Tools (APT) and SNPolisher™ (http://www.affymetrix.com/estore/partners_programs/programs/developer/tools/devnettools.affx (accessed on 22 February 2021)). The objective was to take into account the specificities of the hexaploid wheat genome. For all SNPs, HomRO and HomFLD were calculated. The HomFLD filter was set to 3.6 (http://media.affymetrix.com/support/developer/downloads/Tools/SNPolisher_User_Guide.pdf (accessed on 22 February 2021)). As a first step, all the probesets were processed with a mild inbred penalty equal to 4 on all the samples. As a second step, the SNPs failing the QC criteria (“Other” and “NoMinorHom”) were reprocessed using an inbred penalty of 16. Probesets classified as Off-Target Variants (OTVs) by SNPolisher were analyzed with OTV_caller in the two steps.

Crosses between 19 females with cytoplasmic male sterility [38] and 16 males carrying fertility restorer genes were conducted in an incomplete factorial design and 92 different hybrid combinations were selected for further analyses (Table S2).

### 2.2. Field Experiments

The 92 hybrids, 16 males, and 19 maintainer lines, i.e., isogenic to CMS female lines except for the CMS gene [46], were tested in an augmented design with four replicated checks (Rubisko, RGT Cesario, Tenor and LG Absalon) and a non-replicated check (Chevignon) resulting in a design composed of 160 entries in eight blocks. Because of an insufficient amount of seeds, one hybrid (FEM16 x MA25) was not sown in Arvillers. In addition, due to technical problems, FEM47 female and MA21 male lines were not sown in any location. These two lines were involved in five crosses each and were replaced by the ‘Hyking’ and ‘Hypodrom’ commercial hybrid varieties.

Previous studies performed in five locations in northern France demonstrated that no difference was observed on hybrid yield between a normal and a 15%-reduced sowing density (data not shown). For this reason, hybrids were sown at an 85% density relative to their parents.

Field evaluations were conducted during season 2019/2020 in three locations in France (Table 1). Treatments and fertilization were managed according to the local agricultural practices. In Moinville-la-Jeulin, with a clayey loam type soil, 190 kg N/ha were applied in three applications (respectively 50, 90, and 50 kg N/ha) as well as 40 kg/ha of phosphorous. Two herbicides were applied in autumn. Three fungicide treatments were sprayed in April and May and two insecticides were performed in May. In Arvillers, with a silt limon type soil, 220 kg N/ha in three applications, two herbicides, and three fungicides were applied. In Pomacle, with a chalk type soil, 190 kg N/ha in three applications, one herbicide, and one insecticide before winter and five fungicide treatments were applied. The three nitrogen fertilization were applied during three specific stages: tillering (Z = 26), stem elongation (Z = 30), and booting (Z = 41).

### 2.3. Phenotyping Measurements

For all 92 hybrids, 35 parents, and the checks in all locations, eleven agronomic traits were measured for each plot (Table S3). Grain protein content (GPC) was determined by near infrared spectroscopy using an NIR XDS analyzer (FOSS; Hillerød, Denmark). Two hundred grains were scanned with the MARViN system and analyzed with the MARViN software [47] to estimate grain dimensions (area, width, and length). GPC, thousand kernel weight (TKW), specific weight (SW), and seed dimension data were missing for MA25 and six hybrids in Arvillers (FEM18 x MA22, FEM18 x MA24, FEM09 x MA21, FEM70 x MA25, FEM09 x MA28 and FEM18 x MA08) and two hybrids in Pomacle (FEM09 x MA28 and FEM18 x MA08).

Normalized Difference Vegetation Index (NDVI) was monitored from sowing to harvest with a GreenSeeker^®^ crop sensing system (Trimble, CA, USA). For each plot, between 60 and 80 measurements were performed 50 cm above the canopy. The number of timepoints was on average 13 for Pomacle and 14 for Moinville-la-Jeulin and Arvillers.

### 2.4. Modelling Plant Dynamics

**Figure 1:**
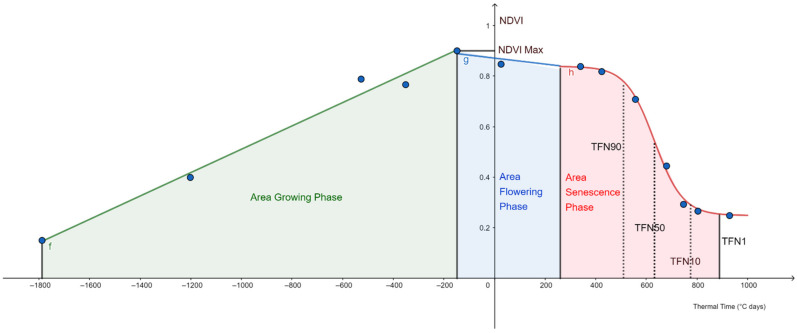
Dynamic changes of wheat vegetation index as a function of thermal time centered on heading date. Example for one wheat line of NDVI modelling based on three phases. NDVI was monitored (blue dots) from sowing to harvest. f, g, and h curves represent the functions calculated in Equations (1)–(3). Descriptions of modeled traits are given in Table S3.

For each genotype, NDVI values were used to establish a plant development model. The developmental cycle was divided in three phases: the “growing phase”, from seedling to maximum NDVI (Nmax); the “flowering phase”, from Nmax to beginning of senescence; and the “senescence phase” from beginning of senescence to end of senescence (Figure 1). NDVI measurement dates were converted into thermal time and expressed in degree days (°C days) with heading date as a reference point (0 °C days) [48]. Thermal time was calculated with a base temperature of 0 °C. For each genotype, three equations were established to fit the three development phases:

(1) $$\mathrm{Growing}\;\mathrm{phase}:\;f\left(TT\right)=a_{1}\;\cdot \;TT+b_{1}$$

(2) $$\mathrm{Flowering}\;\mathrm{phase}:\;g\left(TT\right)=a_{2}\;\cdot \;TT+b_{2}$$

(3) $$\mathrm{Senescence}\;\mathrm{phase}:\;h\left(TT\right)=NDVI_{final}+\frac{NDVI_{amplitude}}{1+e^{a_{3}\;\cdot \;\;TT+b_{3}}}$$

where *a*_1_ and *a*_2_ are the slopes, and *b*_1_ and *b*_2_ the intercepts. *NDVI_final_* is the lowest NDVI value before harvesting and *NDVI_amplitude_* corresponds to the difference between the NDVI at the beginning of senescence and *NDVI_final_*. The exponential term of Equation (3) is composed of a linear model with *a*_3_ being the slope and *b*_3_ the intercept. Thermal time (*TT*) was centered on the heading date (0 °C days). Functions for Equations (1) and (2) were calculated with the “LINEST” Microsoft Excel function. Parameters *a*_3_ and *b*_3_ were determined with the Excel “Solver” add-in using the nonlinear least-squares minimization method.

Several physiological traits linked with stay green were calculated from these functions: slopes, thermal time when 10%, 50%, 90%, and 99% (respectively TFN90, TFN50, TFN10, and TFN1) of *NDVI_amplitude_* was reached [34]. The beginning of the senescence when 90% of the NDVI remained (TFN90) was calculated as $\frac{-\ln\left(9\right)-b_{3}}{a_{3}}$, the mid-senescence (TFN50) was calculated as $\frac{-b_{3}}{a_{3}}$, the inflexion point corresponding to the end of the rapid phase of senescence (TFN10) was calculated as $\frac{\ln\left(9\right)-b_{3}}{a_{3}}$, and the end of the senescence (TFN1) was calculated as $\frac{\ln\left(99\right)-b_{3}}{a_{3}}$. Area under the curve of the growing phase (GPA) was calculated from sowing to thermal time for Nmax. Area under the curve of the “flowering phase” (FPA) was calculated between thermal time for Nmax and thermal time when H(x = 0) where H is the primitive of Equation (3). Area under the curve of the “senescence phase” (SPA) was calculated between thermal time when H(x = 0) and TFN1. We called “area of the declining phase” (DPA) the area between thermal time for Nmax to TNF1 by summing FPA and SPA.

### 2.5. Statistical Analysis

All traits were analyzed for each environment separately with the following fixed ANOVA model for estimating adjusted means:

(4) $$\begin{array}{c} Y_{\mathrm{ij}}\;=\;\mu +\mu_{i}+\beta_{j}+\varepsilon_{\mathrm{ij}}\\ \epsilon {\mathrm{ij}}_{\;}∼\;N(0,\;\sigma_{r}),\;\overset{8}{\underset{1}{\sum }}\beta_{j}=0,\;\overset{133}{\underset{1}{\sum }}\alpha_{i}=0 \end{array}$$

where Y_ij_ is the phenotypic trait for the *i*th genotype in the *j*th block and ε_ij_ the residual error. μ corresponds to the intercept term of the model; α_i_ is the genotypic effect of the *i*th genotype; β_j_ is the *j*th block effect. The best linear unbiased estimators (BLUE) were calculated in R with function “emmeans” from the package “emmeans” for each genotype [49].

All traits were analyzed for each environment separately with the following mixed ANOVA model for calculating heritability:

(5) $$\begin{array}{c} Y_{\mathrm{ij}}\;=\;\mu +\mu_{i}+\beta_{j}+\varepsilon_{\mathrm{ij}}\\ \epsilon {\mathrm{ij}}_{\;}∼\;N(0,\;\sigma_{r}),\;\overset{8}{\underset{1}{\sum }}\beta_{j}=0,\;\alpha_{i}\;∼\;N(0,\;\sigma_{G}) \end{array}$$

where Y_ij_ is the phenotypic trait for the *i*th genotype in the *j*th block and ε_ij_ its residual error. μ corresponds to the intercept term of the model; α_i_ is the random genotypic effect of the *i*th genotype; β_j_ is the *j*th block fixed effect.

Broad sense heritability was calculated as the ratio of genotypic variance to phenotypic variance with the following formula [50]:

(6) $$h^{2}=\frac{\sigma_{G}^{2}}{\sigma_{G}^{2}+\frac{\;\sigma_{r}^{2}}{n}}$$

where $\sigma_{G}^{2}$ corresponds to the genotypic variance, $\sigma_{r}^{2}$ refers to the residual variance, and n is the average number of repetitions.

The traits were also analyzed for estimating adjusted means by including the location effect in a fixed model called “combined environments”:

(7) $$\begin{array}{c} Y_{\mathrm{ijk}}\;=\;\mu +\mu_{i}+\beta_{\mathrm{jk}}+L_{k}+\gamma_{\mathrm{ik}}+\varepsilon_{\mathrm{ijk}}\\ \epsilon {\mathrm{ij}}_{\;}∼\;N(0,\;\sigma_{r}),\;\overset{133}{\underset{1}{\sum }}\alpha_{i}=0,\;\overset{8}{\underset{1}{\sum }}\beta_{jk}=0,\;\overset{3}{\underset{1}{\sum }}L_{k}=0 \end{array}$$

where Y_ijk_ is the phenotypic trait for the *i*th genotype in the *j*th block at *k*th location and ε_ijk_ the residual error. μ corresponds to the intercept term of the model, α_i_ is the genotypic effect of the *i*th genotype, β_jk_ is the *j*th bloc effect at *k*th location, L_k_ is the location effect of the *k*th location, γ_ik_ is the interaction effect for the *i*th genotype at the *k*th location. The best linear unbiased estimators (BLUE) were used in the linear models for estimating the genotypic effect for each genotype.

All traits were analyzed for combined environments with the following mixed ANOVA model for calculating heritability:

(8) $$\begin{array}{c} Y_{\mathrm{ijk}}\;=\;\mu +\mu_{i}+\beta_{\mathrm{jk}}+L_{k}+\gamma_{\mathrm{ik}}+\varepsilon_{\mathrm{ijk}}\\ \epsilon {\mathrm{ijk}}_{\;}∼\;N(0,\sigma_{r}),\;\overset{8}{\underset{1}{\sum }}\beta_{jk}=0,\;\alpha_{i}\;∼\;N(0,\sigma_{G}),\;\overset{3}{\underset{1}{\sum }}L_{k}=0 \end{array}$$

where Y_ijk_ is the phenotypic trait for the *i*th genotype in the *j*th block at *k*th location and ε_ijk_ the residual error. μ corresponds to the intercept term of the model, α_i_ is the genotypic effect of the *i*th genotype, β_jk_ is the *j*th bloc effect at *k*th location, L_k_ is the location effect of the *k*th location, γ_ik_ is the interaction effect for the *i*th genotype at the *k*th location.

Broad sense heritability was calculated as the ratio of genotypic variance to phenotypic variance with the following formulas [50]:

(9) $$h^{2}=\frac{\sigma_{G}^{2}}{\sigma_{G}^{2}+\frac{\sigma_{r}^{2}}{nl}+\frac{\sigma_{\gamma }^{2}}{l}}$$

where $\sigma_{G}^{2}$ corresponds to the genotypic variance,${\;\sigma }_{\gamma }^{2}$ refers to the variance of the interaction, $\sigma_{r}^{2}$ refers to the residual variance, *n* is the average number of repetitions, and *l* is the number of locations.

Pearson coefficient correlations were calculated with the R *cor.test* function. Correlations matrices were plotted in R using the *ggpairs* function from the *GGally* package [51]. Traits were ordered with the following order: measured, estimated, then modelled. Linear models were estimated using the function ‘lm’. For the different performed statistical tests, threshold of significative *p*-value was fixed to α = 0.05.

### 2.6. Heterosis

Heterosis was calculated as the percentage of either mid-parent or best parent heterosis as [52]:

(10) $$\mathrm{Mid}-\mathrm{parent}\;\mathrm{heterosis}:\;Het_{i}=\frac{HYB_{i}-mean(M_{i}+F_{i})}{mean(M_{i}+F_{i})}\times 100$$

and

(11) $$\mathrm{Best}\;\mathrm{parent}\;\mathrm{heterosis}:\;Bet_{i}=\frac{HYB_{i}-\max(M_{i},F_{i})}{max(M_{i},F_{i})}\;\times 100$$

where $HYB_{i}$ is the value for the hybrid *i*, $mean(M_{i}+F_{i})$ is the average between the female and male parents for hybrid *i*, $\max(M_{i},F_{i})$ is the highest value between the female and male parents for the hybrid *i*. No heterosis was calculated for hybrids for which the value for at least one parent was missing.

## 3. Results

### 3.1. Parents and Hybrids Selection

**Figure 2:**
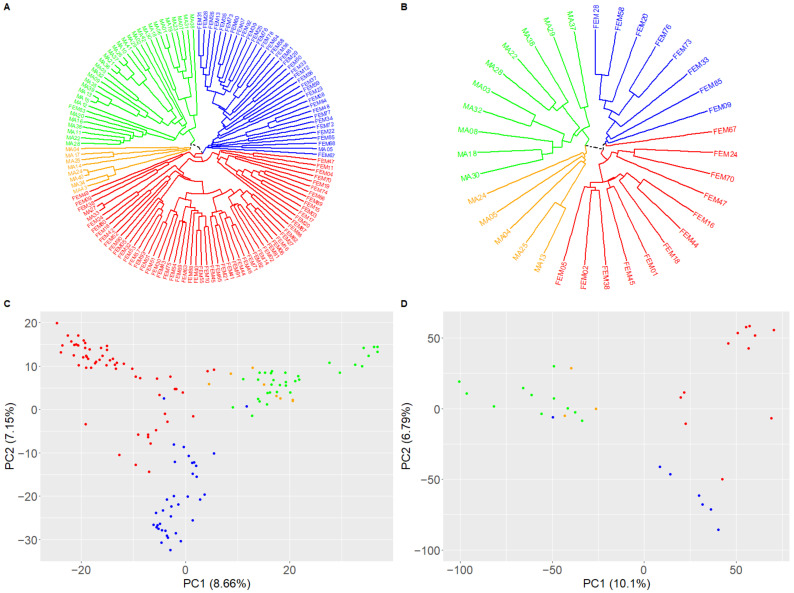
Genetic diversity of the wheat lines. (**A**) Dendrogram showing phylogenetic relationships between 136 wheat lines revealed by 2966 SNP markers (43 males carrying fertility restorer genes in green and orange, 93 females with cytoplasmic male sterility in red and blue). (**B**) Dendrogram showing phylogenetic relationships between 16 males (in green and orange) and 19 females (in red and blue) wheat lines revealed by 214,785 SNP markers. (**C**) Projection of the first and second axes of the principal component analysis based on the dissimilarity matrix of 136 wheat lines calculated with 2966 SNPs (males in green and orange, females in red and blue). (**D**) Projection of the first and second axes of the principal component analysis based on the dissimilarity matrix of 16 males (in green and orange) and 19 females (in red and blue) wheat lines calculated with 214,785 SNPs.

In order to produce a panel of genetically diverse wheat hybrids, we selected a set of 136 wheat lines comprising 43 genotypes carrying fertility restorer genes (Rf), hereafter referred to as ‘males’, and 93 lines carrying a cytoplasmic male sterility (CMS) derived from *Triticum timopheevii* [38], hereafter referred to as ‘females’. These 136 genotypes were genotyped with an Illumina iSelect 15K SNP array [39] and a set of 2966 SNPs was selected to describe the diversity within the genetic panel. The dendrogram revealed four different groups and this clustering was consistent with the projection of the genotypic data onto a principal component analysis (Figure 2A,C). Indeed, the first axis discriminated the males and females while the second axis discriminated females into two groups. Males were divided in two groups on the third axis (data not shown). Eventually, 16 males and 19 females originating from different geographic areas were selected to maximize the genetic diversity within this panel. They were subsequently genotyped with the TaBW410K SNP array and 196,305 PolyHighResolution and 18,480 Off-Target Variant SNPs were selected to analyze the genetic diversity [45]. The dendrogram and the projection of the two first PCA axes of these 35 parent lines showed that diversity was conserved and that four main genetic groups were represented (Figure 2B,D).

Out of the 304 possible female x male combinations, 92 hybrids (30%) were selected for further analyses (Tables S1 and S2). These 92 combinations were chosen in order to have at least three hybrids from each male and two from each female. For males, the average number of hybrid combinations was 5.8 (from 3 to 13) while it was 4.8 (from 2 to 9) for females.

### 3.2. Phenotyping and Modelling

In order to evaluate the physiological and agronomic performances of hybrids, the 92 aforementioned combinations as well as their 35 parents and six checks were sown in three different locations in northern France: Moinville-la-Jeulin, Arvillers, and Pomacle. These three locations were chosen in major wheat production areas and close to the official field trial sites for wheat varieties registration. Average temperatures were similar for the three locations and differences between sowing and harvesting dates did not exceed three weeks. The number of days with high temperature (above 25 °C) was higher in Pomacle (n = 21) than in Arvillers (n = 16) and Moinville-la-Jeulin (n = 13) (Table 1). To characterize the dynamics of the wheat canopy, NDVI was monitored during the whole plant cycle, from emergence to harvest. NDVI data were used to model plant development with the aim to compare similar cycle phases between genotypes. A first phase named “growing phase” was defined with a linear model from sowing date to the NDVI maximum considered as the maximum of plant development. A slightly decreasing plateau modelled with a linear regression was observed after maximum development and was called “flowering phase”. A “senescence phase” was then defined by a sigmoid curve till end of senescence (Figure 1). The “declining phase” included both the “senescence phase” and the “flowering phase”. Due to a lack of measured NDVI points, models were not estimated for five hybrids in Arvillers (FEM18 x MA24, FEM09 x MA21, FEM70 x MA25, FEM09 x MA28 and FEM18 x MA08) and two in Pomacle (FEM09 x MA28 and FEM18 x MA08). For the remaining 466 ‘genotype x location’ combinations, Equations (1)–(3) fitted with measured points with respectively an average R of 0.98, 0.91 and 1.00 and a standard deviation of 0.01, 0.11, and 0.00.

### 3.3. Heritability and Genetic Variation

To estimate the proportion of the variability of each trait accounted for by genetic variance, we calculated heritability in each location as well in the three locations combined, hereafter referred to as ‘combined environments’. Yield heritability was moderate in Moinville-la-Jeulin (h^2^ = 0.57) and Arvillers (h^2^ = 0.66), and high for Pomacle (h^2^ = 0.80) and in the combined environments (h^2^ = 0.69). GPC heritability was moderate to high in all three locations (0.66 to 0.81). Thousand Kernel Weight (TKW) and specific weight (SW) had a high heritability in all environments (0.78–0.97). A similar trend was observed for traits controlled by major genes including plant height and heading date (0.84–0.99). Heritability for the different areas under the curve corresponding to plant phases were environment-dependent and varied from low to high values (0.35–0.92). Regarding the senescence indicators (TFN), their heritability was variable for each individual location (0.35–0.78) except for TFN50 and TFN10 in Moinville-la-Jeulin (0.87 and 0.90) and moderate to high in the three combined environments (0.62–0.82) (Figure 4 and Figures S4–S6).

The fact that heritabilities were high enough for a majority of traits allowed us to calculate adjusted means for each trait in each of the three locations and in the combined environments in order to use these means for trait comparisons and heterosis. The average yield was 8.5 t/ha in the three locations, ranging from 6.6 to 9.8 t/ha (Table 2). Grain protein content ranged from 10.5% to 13.7%, with an average of 11.9%. The average specific weight was above the threshold for superior quality required by the market (81.7 kg/hL), ranging from 78.6 to 84.2 kg/hL. TKW displayed a wide range, from 30.8 to 47.1 g. Similarly, a large diversity was observed for heading date and plant height with 20 days and 44 cm differences between extreme genotypes, respectively. The mean total area modelled from NDVI measurements was in the combined environments of 1314 °C days and variable according to the location with the lowest area in Arvillers (1100 °C days) and the highest in Pomacle (1573 °C days); Moinville-la-Jeulin was intermediary (1276 °C days). The duration of the growing phase was similar between environments (~800 °C days) but displayed a wide range between extreme genotypes (from 727 to 899). The main differences between locations were observed during the declining phase that covers both the flowering and senescence phases with less degree days in Moinville-la-Jeulin (466) compared to Pomacle and Arvillers (respectively 723 and 701) for an average of 627 °C days in the combined environments.

### 3.4. Correlation between Agronomic and Physiological Traits

**Figure 3:**
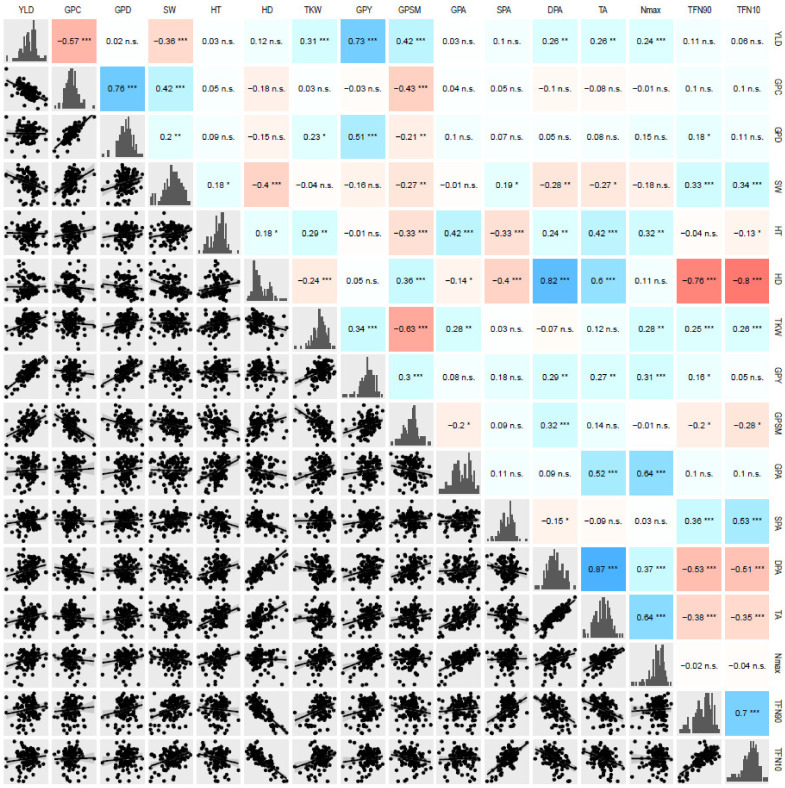
Correlations between adjusted means of phenotypic traits in the combined environments for 92 bread wheat hybrids and their 35 parents. Lower panel represents the linear regression plot. The diagonal shows histogram representing the distribution of each trait. The upper triangle shows Pearson correlations. Positive correlations are colored in blue, negative correlations in red. Significance of the correlation is indicated as either non-significant (n.s.), significant at a *p*-value of 0.05 (*), 0.01 (**), or 0.001 (***). YLD, grain yield; GPC, grain protein content; GPD, grain protein deviation; SW, specific weight; HT, plant height; HD, heading date; TKW, thousand kernel weight; GPY, grain protein yield; GPSM, grains per square meter; GPA, area of the growing phase; SPA, area of the senescence phase; DPA, area of the declining phase area; TA, total NDVI area; Nmax, maximum measured NDVI; TFN90, 90% of the NDVI amplitude remains; TFN10, 10% of the NDVI amplitude remains.

We then calculated correlations between all variables that were either measured or calculated for all locations separately as well as in combined environments (Figure 3 and Figures S1–S3).

There was a strong negative correlation between grain yield and grain protein content (R = −0.57, *p*-value = 2.0 × 10^−10^). As expected, we also found a negative correlation between TKW and the number of grains per square meter (R = −0.63, *p*-value = 2.7 × 10^-11^ ) Finally, stay-green traits were strongly and negatively correlated with heading date (−0.76 to −0.80). A linear model showed that the onset of senescence (TFN90) was significantly correlated to heading date (*p*-value < 2.2 × 10^−16^).

### 3.5. Hybrid Performance and Heterosis

To evaluate the performances of hybrids, we first compared hybrid values to the average values of both parents (mid-parent heterosis).

**Figure 4:**
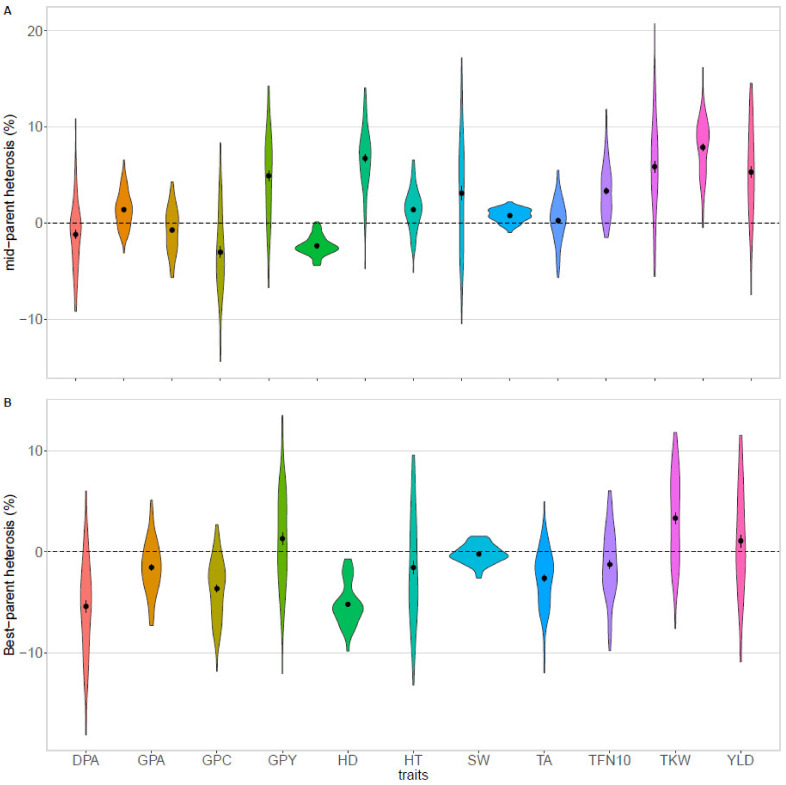
Density distribution of mid-parent (**A**) and best-parent (**B**) heterosis for major traits in the combined environment. Dashed lines correspond to either the mid-parent or the best-parent values. Average heterosis and standard deviation are represented in black dots and bars respectively. DPA, area of the declining phase; GPA, area of the growing phase; GPC, grain protein content; GPY, grain protein yield; HD, heading date; HT, plant height; SW, specific weight; TA, total NDVI area; TFN10, 10% of the NDVI amplitude remains; TKW, thousand kernel weight; YLD, grain yield.

**Figure 5:**
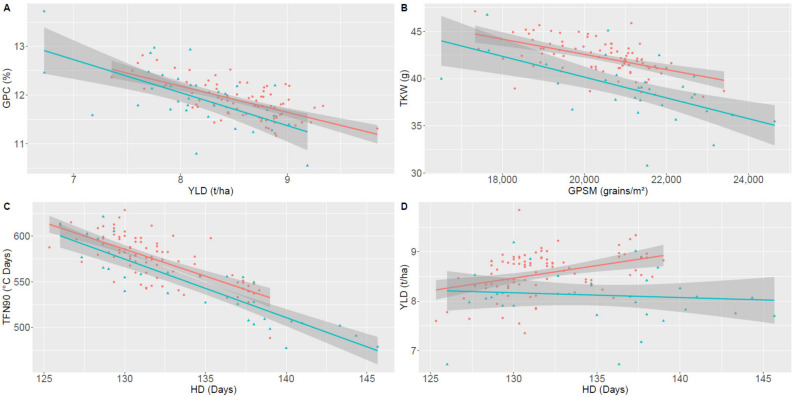
Negative correlations (**A**) between grain yield (YLD) and grain protein content (GPC), (**B**) between grains number per square meter (GPSM) and thousand kernel weight (TKW), (**C**) between heading date (HD) and beginning of the senescence (TFN90), and (**D**) between heading date (HD) and grain yield (YLD) for 92 bread wheat hybrids and their 35 parents studied in three locations. Parent genotypes are represented as blue triangles and hybrids as red circles. The blue and red lines correspond to linear regression models for parents and hybrids, respectively. Shaded areas represent the 95% confidence intervals of the linear regression models.

An average mid-parent heterosis of +5.0% was observed for yield in all environments, with 86% of hybrids performing better than the average of their parents (Table 3, Figure 4A). The maximum was observed in Moinville-la-Jeulin for hybrid FEM85 x MA25 and in Pomacle for hybrid FEM05 x MA25 that both displayed a +23% heterosis. Following this yield increase, hybrids had an average negative heterosis for grain protein content (−0.7%). However, the grain protein deviation was significatively higher for hybrids compared to parent lines leading to +0.21 percentage points increase for hybrids in protein content (*p*-value = 0.04) (Figure 5A). Consequently, the overall protein yield was higher for hybrids than for their mid-parent (+4.7%).

Despite a 15% reduction in sowing density, hybrids had only 3% less grains per square meter at harvest compared to their parents. This decrease was largely compensated by an average +7.7% heterosis for TKW, with 97% of hybrids performing better than their mid-parent in combined environments (Figure 4A, Figures S4A, S5A and S6A). A linear model between TKW and grain per square meter showed that for a given number of grains per square meter, TKW was higher for hybrids compared to pure lines (*p*-value = 9.5 × 10^−8^) (Figure 5B, Figures S7B, S8B and S9B). No significant difference was observed between pure lines and hybrids for the slopes of the negative correlation (*p*-value = 0.2).

The analysis of the plant development dynamics revealed that hybrids grew faster than their parents until the maximal NDVI value (+1.2% heterosis for the area of the growing phase) and had a delayed beginning of senescence (+5.8% for TFN90). In addition, the rate of senescence was lower for hybrids compared to pure lines with a positive heterosis observed for the area of the senescence phase (+3.1%).

Finally, hybrids displayed a negative heterosis for heading date (−2.3%) that translated into heading data that were approximatively 30 °C days earlier.

These tendencies were also observed when only the five higher yielding hybrids were analyzed. Indeed, grain yield heterosis and TKW heterosis was more pronounced for the best hybrids and they were earlier than their parents (Table S11). However, their earliness is less pronounced and having a later heading date than the whole set of hybrids allows them to increase their yield compared to the parental lines (Figure 5D, Figures S7D, S8D and S9D).

Another method to estimate heterosis is to compare hybrids to the best parent line. For this best-parent heterosis, sometimes referred to as heterobeltiosis [53], we did not observe any general tendency for grain yield, grain protein yield, plant height, and TFN90 but rather positive or negative heterosis depending on the location (Table 3 and Tables S8–S10). However, a large variance was observed and for each trait, a number of hybrids over-passing their best parent. For yield, 54% of hybrids were better than both parents with up to +12% heterosis (Figure 4B, Figures S4B, S5B and S6B). Other agronomic and physiological traits were on average lower for hybrids than for the best-parent except TKW and seed area, with respectively +3.6% and +1.5% for combined environments. A significant negative best-parent heterosis was observed in each location for the number of grains per square meter (−7%). The total NDVI area under the curve was smaller for hybrids compared to the best parent with a shorter growing phase due to an earlier heading date and with a shorter declining phase.

## 4. Discussion

Grain yield, grain protein content, and yield stability are three of the most important traits for farmers and the most important targets for breeders. However, not only has wheat yield been stagnating and fluctuating in the main producing countries over the past 20 years, but also grain yield and grain protein content are negatively correlated [54]. Hybrids have been proposed as a way to overcome these limitations by taking advantage of the underexploited wheat heterosis. Indeed, heterosis, i.e., the superiority of the hybrid performance compared to parental lines, has been demonstrated in several species such as maize, sunflower, etc. [13,55,56]. By contrast, the difficulties linked to the use of chemical hybridization agent as well as their limited superiority compared to pure lines have long hampered the development of wheat hybrids. The advent of cytoplasmic male sterile wheat varieties was a game changer and opened the way to the development of better wheat hybrids. However, further insight into wheat heterosis is still needed and, to this aim, we developed an incomplete factorial design composed of 92 hybrids from 35 parent lines and phenotyped them for agronomic traits including yield and yield components as well as for physiological traits.

A positive mid-parent heterosis for grain yield was observed for the vast majority of our hybrids (86%), with an average increase of 5% and up to 23%. In addition, 54% of our combinations displayed a higher grain yield than their best parent, 5% being over the +13% threshold of hybrid profitability compared to pure lines [21]. This result is consistent with previous studies conducted on small or large panels that observed an overall grain yield heterosis for the majority of hybrids [23,29,57,58,59,60] and confirms that inbreeding depression can be efficiently decreased in a self-pollinating species to optimize yield.

As expected from the grain protein concentration-grain yield negative relationship [54], this positive yield heterosis was accompanied with a slightly negative heterosis for grain protein content. However, this very limited negative heterosis (−0.7%) suggested that hybrids were less impacted by the negative correlation. The linear regression between these two traits confirmed this hypothesis. Indeed, while still showing a negative correlation, hybrids showed a positive grain protein deviation (GPD), i.e., the residual from the regression between grain yield and grain protein content [61]. This GPD translated in a +0.21 percentage point increase in protein content, consistent with a previous study conducted on a large panel composed of 1604 hybrids [23].

Overall, this led to a positive heterosis for final protein yield and suggested hybrids broke down the negative balance between high yield and nitrogen metabolism involving less protein in grains [62] resulting in a better nitrogen use efficiency of hybrid genotypes compared to pure lines under the same level of nitrogen fertilization. It seems due to a better nitrogen absorption from the soil for hybrids while the remobilization is similar between hybrids and pure lines [58]. In pure lines, grain protein deviation is highly dependent on the nitrogen absorption after anthesis [63].

To decipher the bases of grain yield heterosis, we analyzed yield component traits. TKW appeared as one of the main explanatory factors and an average positive heterosis of 7.7% was observed, consistent with the results of Askander and collaborators on a small dataset composed of 12 hybrids [28]. However, TKW is known to be negatively correlated with the number of grains per square meter. In our study, hybrids were sown at reduced density compared to pure lines that might have caused this increase in TKW. However, while the reduction in sowing density was 15%, hybrids had only 3% less grains per square meter at harvest compared to their parents, demonstrating that hybrids can compensate for reduced sowing density with increased tillering and spike density, as suggested by Milan et al. [64]. In addition, we also showed that, for a given number of grains per square meter, hybrids had a mean increase of nearly +5% in TKW compared to pure lines, suggesting a more efficient accumulation of resources in the grain.

Besides measuring agronomic traits, we also used NDVI measurements, a non-destructive and heritable method which provides information on leaf area and radiation intercepted by the canopy [65,66]. Monitoring NDVI all along the plant development allowed us to model the different phases and to identify “stay green” varieties, i.e., those with a delayed senescence, a trait that has been shown to impact positively yield in pure lines [34]. We observed a positive heterosis for TFN10, associated with a delayed senescence, that translates into a stay-green phenotype and, subsequently, a longer grain filling time. Nevertheless, despite a normalization of plant cycle in degree days from heading date, stay green traits remained highly impacted by heading date, as reported by Montazeaud et al. and Bogard et al. [35,37]. However, linear models with heading date as covariable showed that hybrids were more susceptible to delayed senescence than the parental lines (Figure 5C, Figures S7C, S8C and S9C). In other words, not only were the hybrids earlier at heading date, but they also finished their senescence later. This finding was supported by the results of several studies that reported stay green QTLs that did not colocalize with genes involved in flowering time or vernalization [67,68,69,70,71]. Combined with a faster grain filling [72,73], the longer filling duration of hybrids is likely responsible for the accumulation of more resources in the grain, resulting in increased TKW and protein yield, and ultimately in a higher yield. Besides its interest for yield increase, early heading date might also help hybrids to escape environmental stresses, such as drought or high temperature, that are expected to become more and more frequent in the coming years, as a result of climate change [74]. The five higher yielding hybrids were intermediate, i.e., later than the average heading date of hybrids and earlier than their parents. They combine the advantages to escape from stressful environments with a longer plant cycle. In this regard, it might be interesting to evaluate this panel over several years to test this hypothesis and assess the effect of heterosis on yield stability.

Finally, while the selection of parental lines relied on SNP-based genetic distances, it would be interesting to characterize the 16 male and 19 female lines for genomic structural variations (SVs). Indeed, it is now widely admitted that SVs play a role in hybrid vigor through the combination of complementary gene and allele contents [75,76]. While in maize, hybrids result from crosses between different heterotic groups, no apparent structure was observed in the registered wheat varieties [77]. Despite this lack of heterotic groups, many wheat hybrids out-perform their parents. Combining additive-dominance effects with copy variants, as well as better knowledge of the best pairing of fertility restoration genes, has the potential to improve the prediction of the best-performing hybrids.

## 5. Conclusions

Hybrid wheat produced with chemical hybridization agent has been widely studied over the past decades. Unfortunately, with this technique, the production of hybrids is fastidious and difficult. The new generation of hybrids produced by cytoplasmic male sterility has helped to decrease these difficulties and to increase the number of combinations tested. Our study focused on heterosis in 92 CMS hybrid combinations compared to their 35 parents. It appears that a majority of hybrids have a mid-parent heterosis for grain yield with a very limited decrease in grain protein content thanks to a positive GPD. Grain yield heterosis is made possible thanks to a positive heterosis on the thousand kernel weight due to a longer filling time for hybrids.

## Figures and Tables

**Table 1:** Key characteristics of the three field trial sites in France where bread wheat experiments were conducted.

Location	Coordinates	Plot Size (m^2^)	Sowing Date	Harvest Date	Cycle Duration(Days)	Average Air Temperature (°C)	Sum of Temperatures (°C Days)	Number of Days <0 °C	Number of Days >25 °C	Cumulative Rainfall (mm)
Moinville-la-Jeulin	Lat: 48.38, Lon: 1.7	12.4	25/10/2019	11/07/2020	260	10.4	2709	35	13	347
Arvillers	Lat: 49.73, Lon: 2.65	10.6	29/10/2019	29/07/2020	274	10.4	2854	39	16	382
Pomacle	Lat: 49.33, Lon: 4.15	12.0	12/10/2019	19/07/2020	281	10.5	2953	47	21	430

**Table 2:** Descriptive statistics of phenotypic traits measured on 92 wheat hybrids and their 35 parents studied in three locations in northern France. Average, minimal, and maximal values as well as heritabilities (h^2^), genetic variances (V_α_), and residual errors (V_ε_) were calculated for all phenotypic traits.

Trait	Unit	Mean	Min	Max	H^2^	V_α_	V_γ_	V_ε_
YLD	t/ha	8.5	6.6	9.8	0.69	4.4 × 10^−1^	3.7 × 10^−1^	3.9 × 10^−1^
GPC	%	11.9	10.5	13.7	0.81	4.0 × 10^−1^	2.0 × 10^−1^	3.0 × 10^−1^
SW	kg/hL	81.7	78.6	84.2	0.87	1.1 × 10^0^	6.1 × 10^−1^	4.7 × 10^−1^
HD	days	132.6	125.3	145.7	0.97	3.9 × 10^0^	9.6 × 10^−1^	6.6 × 10^−1^
HT	cm	93.4	72.0	116.0	0.95	7.1 × 10^0^	0.0 × 10^0^	3.1 × 10^0^
SEEDA	mm^2^	16.0	12.6	18.4	0.94	8.9 × 10^−1^	2.6 × 10^−1^	3.3 × 10^−1^
SEEDL	mm	6.3	5.5	7.0	0.95	2.2 × 10^−1^	5.6 × 10^−2^	6.7 × 10^−2^
SEEDW	mm	3.5	3.2	3.7	0.87	9.0 × 10^−2^	4.0 × 10^−2^	4.8 × 10^−2^
TKW	g	41.1	30.8	47.1	0.84	2.9 × 10^0^	2.0 × 10^0^	8.2 × 10^−1^
GPY	t/ha	1.0	0.8	1.1	0.48	3.5 × 10^−2^	4.6 × 10^−2^	4.7 × 10^−2^
GPSM	grains/m^2^	20,614.9	16,514.2	24,633.9	0.76	1.3 × 10^3^	9.0 × 10^2^	9.5 × 10^2^
Nmax	-	0.9	0.8	0.9	0.68	1.9 × 10^−2^	1.7 × 10^−2^	1.6 × 10^−2^
GPA	-	834.8	727.8	899.0	0.88	3.2 × 10^1^	1.2 × 10^1^	1.8 × 10^1^
FPA	-	339.7	257.4	467.3	0.87	4.3 × 10^1^	1.9 × 10^1^	2.3 × 10^1^
SPA	-	287.7	230.2	342.2	0.35	1.0 × 10^1^	1.7 × 10^1^	1.8 × 10^1^
DPA	-	627.4	530.7	732.0	0.83	3.5 × 10^1^	2.0 × 10^1^	2.1 × 10^1^
TA	-	1314.7	1138.4	1443.8	0.75	4.3 × 10^1^	3.2 × 10^1^	3.0 × 10^1^
TFN90	°C days	567.2	477.4	628.7	0.71	2.6 × 10^1^	2.7 × 10^1^	1.4 × 10^1^
TFN50	°C days	688.5	592.2	742.5	0.82	2.8 × 10^1^	1.7 × 10^1^	1.7 × 10^1^
TFN10	°C days	809.8	704.0	889.2	0.76	3.0 × 10^1^	1.7 × 10^1^	2.6 × 10^1^
TFN1	°C days	942.2	820.3	1062.6	0.62	3.2 × 10^1^	1.6 × 10^1^	4.4 × 10^1^

YLD, grain yield; GPC, grain protein content; SW, specific weight; HD, heading date; HT, plant height; SEEDA, seed area; SEEDL, seed length; SEEDW, seed width; TKW, thousand kernel weight; GPY, grain protein yield; GPSM, grains per square meter; Nmax, maximum measured NDVI; GPA, area of the growing phase; FPA, area of the flowering phase; SPA, area of the senescence phase; DPA, area of the declining phase; TA, total NDVI area; TFN90, 90% of the NDVI amplitude remains; TFN50, 50% of the NDVI amplitude remains; TFN10, 10% of the NDVI amplitude remains; TFN1, 1% of the NDVI amplitude remains.

**Table 3:** Mid-parent and best-parent heterosis of wheat F_1_ hybrids studied in three locations. For each trait, the number of hybrids tested, the average heterosis, the *p*-value of the Student test between hybrids and parent lines, the minimal, and maximal values, as well as the heterosis coefficient of variation are indicated.

		Mid-Parent Heterosis					Best-Parent Heterosis				
Trait	Number of Hybrids	Mean (%)	*p*-Value	Min (%)	Max (%)	σ/μ	Mean (%)	*p*-Value	Min (%)	Max (%)	σ/μ
YLD	80	5.0	3.2 × 10^−12^	−16.4	14.6	1.1	0.9	n.s	−17.7	11.6	6.6
GPC	75	−0.7	2.2 × 10^−2^	−5.7	4.4	−3.7	−3.6	2.2 × 10^−15^	−11.8	2.7	−0.9
SW	75	0.8	4.5 × 10^−15^	−1.0	2.2	0.9	−0.2	3.8 × 10^−2^	−2.6	1.5	−4.1
HD	80	−2.3	3.7 × 10^−34^	−4.4	0.1	−0.4	−5.2	4.5 × 10^−35^	−9.8	−0.7	−0.4
HT	80	6.7	1.3 × 10^−29^	−4.7	14.1	0.5	−1.5	1.4 × 10^−2^	−13.2	9.6	−3.6
SEEDA	74	5.6	5.0 × 10^−23^	−6.9	13.8	0.6	1.5	3.6 × 10^−3^	−13.4	9.8	2.8
SEEDL	74	2.0	2.6 × 10^−18^	−2.1	5.1	0.7	−0.7	1.5 × 10^−2^	−7.2	3.6	−3.4
SEEDW	74	3.3	5.8 × 10^−23^	−5.1	7.8	0.6	1.5	1.3 × 10^−6^	−6.6	7.3	1.6
TKW	75	7.7	1.2 × 10^−26^	−6.6	16.2	0.5	3.6	5.2 × 10^−6^	−14.7	12.6	1.4
GPY	75	4.7	2.4 × 10^−12^	−9.7	14.3	1.0	1.2	4.9 × 10^−2^	−12.1	13.5	4.3
GPSM	66	−3.1	7.3 × 10^−7^	−14.4	8.3	−1.5	−7.0	5.1 × 10^−19^	−18.5	2.8	−0.6
Nmax	75	1.2	1.2 × 10^−4^	−10.7	6.6	2.1	−0.6	n.s	−12.6	3.9	−4.5
GPA	75	1.2	4.0 × 10^−5^	−11.7	6.6	2.0	−1.7	3.3 × 10^−6^	−11.9	5.1	−1.7
FPA	75	−4.6	4.3 × 10^−6^	−21.8	17.4	−1.7	−13.7	1.2 × 10^−17^	−33.6	10.6	−0.8
SPA	75	3.1	3.1 × 10^−5^	−10.5	17.2	1.9	−2.8	2.5 × 10^−4^	−16.2	12.2	−2.2
DPA	75	−1.3	4.3 × 10^−3^	−10.7	10.9	−2.9	−5.5	2.1 × 10^−14^	−18.2	6.0	−0.9
TA	75	0.1	n.s	−11.4	5.5	26.8	−2.8	1.6 × 10^−10^	−13.7	5.0	−1.2
TFN90	75	5.8	7.9 × 10^−15^	−5.6	20.7	0.9	0.1	n.s	−13.8	11.5	50.1
TFN50	75	4.3	1.5 × 10^−18^	−2.7	12.1	0.7	−0.7	n.s	−11.1	6.5	−5.8
TFN10	75	3.4	6.3 × 10^−16^	−1.5	11.8	0.8	−1.3	2.6 × 10^−3^	−9.8	6.1	−2.8
TFN1	75	2.6	2.9 × 10^−9^	−4.7	11.6	1.3	−1.8	1.6 × 10^−4^	−11.5	8.1	−2.2

YLD, grain yield; GPC, grain protein content; SW, specific weight; HD, heading date; HT, plant height; SEEDA, seed area; SEEDL, seed length; SEEDW, seed width; TKW, thousand kernel weight; GPY, grain protein yield; GPSM, grains per square meter; Nmax, maximum NDVI measured; GPA, area of the growing phase; FPA, area of the flowering phase; SPA, area of the senescence phase; DPA, area of the declining phase; TA, total NDVI area; TFN90, 90% of the NDVI amplitude remains; TFN50, 50% of the NDVI amplitude remains; TFN10, 10% of the NDVI amplitude remains; TFN1, 1% of the NDVI amplitude remains. n.s: non-significant.

## Data Availability

The data presented in this study are available in the Supplementary Materials.

## Supplementary Materials

The following are available online at https://www.mdpi.com/article/10.3390/biology10090907/s1, Table S1: Best Linear Unbiased Estimators (BLUEs) for each environment. Table S2: correspondence between the line names of the wheat genotypes and the code names. Table S3: Incomplete factorial bread wheat F1 hybrid mating design: Females (FEM; in blue and red) in rows and males (MA; in orange and green) in columns. “X” indicates hybrids that have been tested in this study. Colors corresponds to genetic groups identified in Figure 2. Table S4: Description of phenotypic traits. Table S5: Descriptive statistics of phenotypic traits in Moinville-la-Jeulin. Average, minimal, and maximal values as well as heritabilities (H^2^), genetic variances (Vα), and residual errors (Vε) were calculated for all phenotypic traits. Table S6: Descriptive statistics of phenotypic traits in Arvillers. Average, minimal, and maximal values as well as heritabilities (H^2^), genetic variances (Vα), and residual errors (Vε) were calculated for all phenotypic traits. Table S7: Descriptive statistics of phenotypic traits in Pomacle. Average, minimal, and maximal values as well as heritabilities (H^2^), genetic variances (Vα) and residual errors (Vε) were calculated for all phenotypic traits. Table S8: Mid-parent and best-parent heterosis of F1 hybrids in Moinville-la-Jeulin. For each trait, the number of hybrids tested, the average heterosis, the *p*-value of the Student test between hybrids and parent lines, the minimal and maximal value, as well as the heterosis coefficient of variation are indicated. Table S9: Mid-parent and Best-parent heterosis of F1 hybrids in Arvillers. For each trait, the number of hybrids tested, the average heterosis, the *p*-value of the Student test between hybrids and parent lines, the minimal and maximal value, as well as the heterosis coefficient of variation are indicated. Table S10: Mid-parent and Best-parent heterosis of F1 hybrids in Pomacle. For each trait, the number of hybrids tested, the average heterosis, the *p*-value of the Student test between hybrids and parent lines, the minimal and maximal value, as well as the heterosis coefficient of variation are indicatedTable S11: Mid-parent and Best-parent heterosis of the five best F1 hybrids for yield in the three locations.. Figure S1. Correlations between adjusted means of phenotypic traits in Moinville-la-Jeulin. Lower panel represents the linear regression plot. The diagonal shows histogram representing the distribution of each trait. The upper triangle shows Pearson correlations. Positive correlations are colored in blue, negative correlations in red. Significance of the correlation is indicated as either non-significant (n.s.), significant at a *p*-value of 0.05 (*), 0.01 (**), or 0.001 (***).Figure S2. Correlations between adjusted means of phenotypic traits in Arvillers. Lower panel represents the linear regression plot. The diagonal shows histogram representing the distribution of each trait. The upper triangle shows Pearson correlations. Positive correlations are colored in blue, negative correlations in red. Significance of the correlation is indicated as either non-significant (n.s.), significant at a *p*-value of 0.05 (*), 0.01 (**), or 0.001 (***).Figure S3. Correlations between adjusted means of phenotypic traits in Pomacle. Lower panel represents the linear regression plot. The diagonal shows histogram representing the distribution of each trait. The upper triangle shows Pearson correlations. Positive correlations are colored in blue, negative correlations in red. Significance of the correlation is indicated as either non-significant (n.s.), significant at a *p*-value of 0.05 (*), 0.01 (**), or 0.001 (***).Figure S4. Density distribution of mid-parent (A) and best-parent (B) heterosis for major traits in Moinville-la-Jeulin. Dashed lines correspond to either the mid-parent or the best-parent values. Average heterosis and standard deviation are represented in black dots and bars respectively. Figure S5. Density distribution of mid-parent (A) and best-parent (B) heterosis for major traits in Arvillers. Dashed lines correspond to either the mid-parent or the best-parent values. Average heterosis and standard deviation are represented in black dots and bars respectively. Figure S6. Density distribution of mid-parent (A) and best-parent (B) heterosis for major traits in Pomacle. Dashed lines correspond to either the mid-parent or the best-parent values. Average heterosis and standard deviation are represented in black dots and bars respectively. Figure S7. Negative correlations (A) between grain yield (YLD) and grain protein content (GPC), (B) between grains number per square meter (GPSM) and thousand kernel weight (TKW), (C) between heading date (HD) and beginning of the senescence (TFN90) and (D) between heading date (HD) and grain yield (YLD) for 92 bread wheat hybrids and their 35 parents studied in Moinville-la-Jeulin. Parent genotypes are represented as blue triangles and hybrids as red circles. The blue and red lines correspond to linear regression models for parents and hybrids, respectively. Shaded areas represent the 95% confidence intervals of the linear regression models. Figure S8. Negative correlations (A) between grain yield (YLD) and grain protein content (GPC), (B) between grains number per square meter (GPSM) and thousand kernel weight (TKW), (C) between heading date (HD) and beginning of the senescence (TFN90) and (D) between heading date (HD) and grain yield (YLD) for 92 bread wheat hybrids and their 35 parents experimentally studied in Arvillers. Parent genotypes are represented as blue triangles and hybrids as red circles. The blue and red lines correspond to linear regression models for parents and hybrids, respectively. Shaded areas represent the 95% confidence intervals of the linear regression models. Figure S9. Negative correlations (A) between grain yield (YLD) and grain protein content (GPC), (B) between grain number per square meter (GPSM) and thousand kernel weight (TKW), (C) between heading date (HD) and beginning of the senescence (TFN90), and (D) between heading date (HD) and grain yield (YLD) for 92 bread wheat hybrids and their 35 parents experimentally studied in Pomacle. Parent genotypes are represented as blue triangles and hybrids as red circles. The blue and red lines correspond to linear regression models for parents and hybrids, respectively. Shaded areas represent the 95% confidence intervals of the linear regression models.
