# Supplementary material for: Dissecting Bread Wheat Heterosis through the Integration of Agronomic and Physiological Traits
Source: Biology (Basel). 2021 Sep 13;10(9):907. doi: 10.3390/biology10090907 (PMC8465846; doi:10.3390/biology10090907)
Supplement: Supplementary file 1 [file biology-10-00907-s001.zip › supplemental data/Supplementary Table.pdf]

Supplementary Data

Table S2: correspondence between the line names of the wheat genotypes and the code names.

Line name	Code name
TW16TX020472	FEM01
TW16TX020386	FEM02
H16FR15042	FEM03
TW16TX020251	FEM04
TW16TX030811	FEM05
H13FR14273	FEM06
H16FR14997	FEM07
TW16TX020212	FEM08
H1144033	FEM09
TW16TX020444	FEM10
H15FR14199	FEM11
H15FR13965	FEM12
TW16TX020166	FEM13
TW16TX020391	FEM14
H16FR14826	FEM15
TW16TX030804	FEM16
H15FR14855	FEM17
H13FR14654	FEM18
TW16TX020287	FEM19
H15FR15131	FEM20
TW16TX020338	FEM21
TW16TX020148	FEM22
H1111183	FEM23
TW16TX020402	FEM24
H16FR14992	FEM25
H16FR15036	FEM26
SYN 10739	FEM27
H06334	FEM28
TW16TX030806	FEM29
TW16TX020500	FEM30
TW16TX020254	FEM31
TW16TX030810	FEM32
TW16TX020197	FEM33
TW16TX020475	FEM34

H16FR15187	FEM35
H16FR14978	FEM36
TW16TX020372	FEM37
SY 116421	FEM38
H16FR15024	FEM39
TW16TX020505	FEM40
TW16TX030801	FEM41
H13FR14276	FEM42
TW16TX020503	FEM43
TW16TX023062	FEM44
TW16TX020459	FEM45
TW16TX023061	FEM46
H10611	FEM47
TW16TX020106	FEM48
H1111265	FEM49
TW16TX020191	FEM50
TW16TX020451	FEM51
H15FR14285	FEM52
TW16TX020421	FEM53
TW16TX020510	FEM54
TW16TX020454	FEM55
TW16TX020375	FEM56
TW16TX023069	FEM57
H16FR15162	FEM58
TW16TX020163	FEM59
H16FR15015	FEM60
H14FR13664	FEM61
TW16TX030809	FEM62
TW16TX020501	FEM63
H16FR14980	FEM64
TW16TX030812	FEM65
H15FR15113	FEM66
TW16TX020008	FEM67
H15FR14333	FEM68
TW16TX020441	FEM69
TW16TX020310	FEM70
TW16TX030803	FEM71
TW16TX020146	FEM72
H16FR15007	FEM73
H15FR15114	FEM74
TW16TX020430	FEM75
H16FR15017	FEM76
TW16TX020119	FEM77
H16FR14971	FEM78

ROBIGUS	FEM79
TW16TX020404	FEM80
TW16TX020492	FEM81
TW16TX020493	FEM82
H1111142	FEM83
TW16TX020126	FEM84
H16FR15038	FEM85
TW16TX020423	FEM86
TW16TX020471	FEM87
TW16TX021726	FEM88
H15FR15060	FEM89
TW16TX023060	FEM91
H16FR14999	FEM92
TW16TX020424	FEM93
RTW17TX020100	MA01
RTW17TX020234	MA02
RTW17TX020253	MA03
RTW16TX010075	MA04
RTW16TX010251-1	MA05
RTW17TX020066	MA06
RTW17TX020152	MA07
RTW16TX020050	MA08
RTW16TX030004-1	MA09
RTW16TX010029	MA10
R16FR14053	MA11
RTW17TX020357	MA12
RTW16TX010249-1	MA13
RTW16TX010116	MA14
RTW16TA003043-4	MA15
R16FR14021	MA16
RTW16TX010010-1	MA17
RTW17TX020009	MA18
RTW17TX020047	MA19
R16FR14050	MA20
RTW16TX010030	MA21
R16FR14045	MA22
RTW17TX020077	MA23
RTW16TX010093	MA24
RTW16TX010036-2	MA25
RTW16TX010035	MA26
RTW16TX010043	MA27
R16FR14130	MA28
RTW16TX010244-2	MA29
RTW17TX020121	MA30

RTW17TX020011	MA31
RTW17TX020270	MA32
RTW16TX010044	MA33
RTW16TX010260	MA34
RTW16TX030054	MA35
RTW16TX010033	MA36
RTW16TX010026	MA37
R16FR14032	MA38
RTW16TA003042	MA39
RTW16TX010095	MA40
RTW16TX010054-2	MA41
RTW17TX020209	MA42
RTW16TX010061-1	MA43

Table S3: Incomplete factorial bread wheat F1 hybrid mating design: Females (FEM; in blue and red) in rows and males (MA; in orange and green) in columns. "X" indicates hybrids that have been tested in this study. Colors corresponds to genetic groups identified in Figure 2.

	MA24	MA05	MA04	MA25	MA13	MA22	MA28	MA18	MA30	MA38	MA03	MA37	MA21	MA29	MA08	MA32	Total
FEM09	X		X		X		X		X			X	X	X			8
FEM85				X		X				X							3
FEM33				X		X											2
FEM73	X	X					X				X	X	X		X	X	8
FEM76			X		X	X						X	X		X		6
FEM20	X	X	X		X		X	X				X		X			8
FEM44				X		X				X							3
FEM28	X							X	X	X	X	X	X		X		7
FEM02				X		X											2
FEM05				X		X		X		X							4
FEM16				X		X				X							3
FEM18	X		X		X	X		X			X	X	X		X		9
FEM24				X		X											2
FEM38				X		X				X				X			4
FEM45				X		X			X	X							4
FEM47		X			X		X		X							X	5
FEM58					X				X	X		X		X	X	X	7
FEM67				X		X				X							3
FEM70				X		X				X							3
Total	5	3	4	11	6	13	4	4	5	10	3	7	5	4	5	3	92

Table S4: Description of phenotypic traits

Trait	Unit	Abbreviation	Description
Heading date	days	HD	50% of the ears emerged at 100%
Plant height	cm	HT	measured from the ground until the top of the canopy
Grain protein content	%	GPC	protein concentration estimated with a NIR XDS analyser (FOSS; Denmark).
Seed area	mm^2	SEEDA	mean of the surface for 100 seeds estimated with a MARViN system
Seed length	mm	SEEDL	mean of the length for 100 seeds estimated with a MARViN system
Seed width	mm	SEEDW	mean of the width for 100 seeds estimated with a MARViN system
Specific weight	kg/hL	SW	weight of 1000 grains divided by its volume
Thousand kernel weight	g	TKW	weight of 1000 grains normalized at 0% of humidity
Grain yield	t/ha	YLD	weight of grains harvested normalized at 0% of humidity
Grain protein deviation	-	GPD	residuals from the regression of grain protein content on grain yield
Grain protein yield	t/ha	GPY	estimated by multiplying grain yield by grain protein content
Grains per square meter	grain/m^2	GPSM	estimated by dividing grain yield by thousand kernel weight
NDVI MAX	-	Nmax	maximum of measured NDVI
Area of the growing phase	-	GPA	integration from sowing to Nmax of the linear model of NDVI as a function of thermal time
Area of the flowering phase	-	FPA	integration from Nmax to the beginning of senescence of the linear model of NDVI as a function of thermal time
Area of the senescence phase	-	SPA	integration of the logistic model from primitive H(x=0) of Equation 3 to end of senescence (TFN1)
Area of the declining phase	-	DPA	sum of area of the flowering phase and area of the senescence phase
Total NDVI area	-	TA	sum of area of the growing phase, area of the flowering phase and area of the senescence phase
TFN90	°C days	TFN90	thermal time when 90% of the NDVI amplitude remains
TFN50	°C days	TFN50	thermal time when 50% of the NDVI amplitude remains
TFN10	°C days	TFN10	thermal time when 10% of the NDVI amplitude remains
TFN1	°C days	TFN1	thermal time when 1% of the NDVI amplitude remains

Table S5: Descriptive statistics of phenotypic traits in Moinville-la-Jeulin. Average, minimal and maximal values as well as heritabilities (H^2), genetic variances (V_α) and residual errors (V_ε) were calculated for all phenotypic traits.

Trait	Unit	Mean	Min	Max	H^2	V_α	V_ε
YLD	t/ha	7.0	5.3	8.4	0.57	1.7E-01	1.5E-01
GPC	%	12.1	10.2	14.6	0.66	2.4E-01	1.5E-01
SW	kg/hL	83.9	79.7	87.3	0.78	1.5E+00	5.2E-01
HD	days	127.5	121.0	141.0	0.98	1.5E+01	4.2E-01
HT	cm	91.1	67.0	116.0	0.91	5.1E+01	6.2E+00
SEEDA	mm^2	16.2	12.9	18.6	0.82	7.2E-01	1.9E-01
SEEDL	mm	6.3	5.5	6.9	0.73	8.3E-03	3.6E-03
SEEDW	mm	3.6	3.2	3.8	0.93	4.5E-02	4.3E-03
TKW	g	42.4	32.1	50.1	0.92	8.7E+00	8.9E-01
GPY	t/ha	0.8	0.7	1.0	0.54	1.5E-03	1.5E-03
GPSM	$grains/m^2$	16539.5	12672.0	20448.9	0.56	1.2E+06	1.1E+06
Nmax	-	0.9	0.7	0.9	0.78	8.4E-04	2.9E-04
GPA	-	810.0	701.5	893.4	0.66	1.1E+03	6.7E+02
FPA	-	205.3	70.4	349.8	0.87	2.5E+03	4.6E+02
SPA	-	260.7	204.5	316.9	0.87	4.6E+02	8.0E+01
DPA	-	466.0	347.4	570.7	0.71	1.0E+03	5.0E+02
TA	-	1276.0	1118.3	1463.9	0.64	2.2E+03	1.5E+03
TFN90	°C days	584.7	427.1	694.1	0.43	7.9E+02	1.3E+03
TFN50	°C days	665.1	547.2	736.3	0.87	1.6E+03	2.7E+02
TFN10	°C days	745.6	621.4	832.3	0.90	2.0E+03	2.7E+02
TFN1	°C days	833.3	678.2	946.7	0.59	1.9E+03	1.6E+03

YLD, grain yield; GPC, grain protein content; SW, specific weight; HD, heading date; HT, plant height; SEEDA, seed area; SEEDL, seed length; SEEDW, seed width; TKW, thousand kernel weight; GPY, grain protein yield; GPSM, grains per square meter; Nmax, maximum measured NDVI; GPA, area of the growing phase; FPA, area of the flowering phase; SPA, area of the senescence phase; DPA, area of the declining phase; TA, total NDVI area; TFN90, 90% of the NDVI amplitude remains; TFN50, 50% of the NDVI amplitude remains; TFN10, 10% of the NDVI amplitude remains; TFN1, 1% of the NDVI amplitude remains

Table S6: Descriptive statistics of phenotypic traits in Arvillers. Average, minimal and maximal values as well as heritabilities (H^2), genetic variances (V_α) and residual errors (V_ε) were calculated for all phenotypic traits.

Trait	Unit	Mean	Min	Max	H^2	V_α	V_ε
YLD	t/ha	10.4	8.0	12.2	0.66	2.9E-01	1.8E-01
GPC	%	12.3	11.3	13.6	0.78	1.7E-01	5.6E-02
SW	kg/hL	80.2	76.3	82.8	0.94	1.8E+00	1.3E-01
HD	days	138.7	130.0	151.0	0.97	1.5E+01	6.1E-01
HT	cm	98.6	74.0	123.0	0.84	6.0E+01	1.4E+01
SEEDA	mm^2	16.0	12.7	18.1	0.91	8.6E-01	9.5E-02
SEEDL	mm	6.4	5.6	7.0	0.88	1.3E-02	2.1E-03
SEEDW	mm	3.5	3.1	3.8	0.88	5.0E-02	7.9E-03
TKW	g	40.0	30.2	46.5	0.97	2.1E+01	8.2E-01
GPY	t/ha	1.3	1.0	1.5	0.54	3.4E-03	3.4E-03
GPSM	grains/m^2	25800.3	21076.4	30971.4	0.77	2.7E+06	9.9E+05
Nmax	-	0.9	0.8	1.0	0.68	2.5E-04	1.4E-04
GPA	-	843.4	730.5	908.4	0.84	9.5E+02	2.1E+02
FPA	-	420.7	280.4	624.2	0.78	2.5E+03	8.5E+02
SPA	-	280.7	176.4	338.7	0.50	4.0E+02	4.6E+02
DPA	-	701.4	559.8	801.7	0.89	1.7E+03	2.5E+02
TA	-	1102.3	917.4	1216.2	0.89	2.3E+03	3.4E+02
TFN90	°C days	588.6	479.9	675.3	0.61	7.1E+02	5.3E+02
TFN50	°C days	740.5	630.9	806.2	0.78	8.0E+02	2.7E+02
TFN10	°C days	892.5	755.1	1016.7	0.54	8.5E+02	8.7E+02
TFN1	°C days	1058.3	869.0	1264.5	0.35	1.1E+03	2.4E+03

YLD, grain yield; GPC, grain protein content; SW, specific weight; HD, heading date; HT, plant height; SEEDA, seed area; SEEDL, seed length; SEEDW, seed width; TKW, thousand kernel weight; GPY, grain protein yield; GPSM, grains per square meter; Nmax, maximum measured NDVI; GPA, area of the growing phase; FPA, area of the flowering phase; SPA, area of the senescence phase; DPA, area of the declining phase; TA, total NDVI area; TFN90, 90% of the NDVI amplitude remains; TFN50, 50% of the NDVI amplitude remains; TFN10, 10% of the NDVI amplitude remains; TFN1, 1% of the NDVI amplitude remains

Table S7: Descriptive statistics of phenotypic traits in Pomacle. Average, minimal and maximal values as well as heritabilities (H^2), genetic variances (V_α) and residual errors (V_ε) were calculated for all phenotypic traits.

Trait	Unit	Mean	Min	Max	H^2	V_α	V_ε
YLD	t/ha	8.2	5.7	10.0	0.80	5.0E-01	1.5E-01
GPC	%	11.3	10.1	13.2	0.72	1.7E-01	7.8E-02
SW	kg/hL	80.9	76.4	83.1	0.97	1.5E+00	5.1E-02
HD	days	131.8	124.0	145.0	0.99	1.9E+01	2.6E-01
HT	cm	91.0	73.0	123.0	0.92	4.9E+01	5.2E+00
SEEDA	mm^2	15.9	12.1	18.9	0.96	1.0E+00	4.7E-02
SEEDL	mm	6.2	5.4	7.1	0.92	1.1E-02	1.0E-03
SEEDW	mm	3.5	3.1	3.9	0.98	6.1E-02	1.2E-03
TKW	g	41.1	30.1	47.9	0.97	8.1E+00	3.1E-01
GPY	t/ha	0.9	0.7	1.1	0.77	4.9E-03	1.8E-03
GPSM	$grains/m^2$	19524.6	13748.9	23391.2	0.84	3.2E+06	7.1E+05
Nmax	-	0.9	0.7	0.9	0.72	8.7E-04	4.0E-04
GPA	-	850.4	692.8	921.2	0.92	1.4E+03	1.4E+02
FPA	-	410.0	296.5	569.3	0.79	1.2E+03	3.8E+02
SPA	-	313.3	222.7	412.1	0.51	3.4E+02	4.0E+02
DPA	-	723.3	600.4	884.1	0.81	2.1E+03	6.0E+02
TA	-	1573.7	1305.9	1764.1	0.83	4.2E+03	1.0E+03
TFN90	°C days	524.7	400.1	594.1	0.69	9.4E+02	4.9E+02
TFN50	°C days	658.2	553.7	736.7	0.72	8.2E+02	3.9E+02
TFN10	°C days	791.7	669.5	901.1	0.59	8.8E+02	7.4E+02
TFN1	°C days	937.4	785.7	1109.5	0.43	1.1E+03	1.7E+03

YLD, grain yield; GPC, grain protein content; SW, specific weight; HD, heading date; HT, plant height; SEEDA, seed area; SEEDL, seed length; SEEDW, seed width; TKW, thousand kernel weight; GPY, grain protein yield; GPSM, grains per square meter; Nmax, maximum measured NDVI; GPA, area of the growing phase; FPA, area of the flowering phase; SPA, area of the senescence phase; DPA, area of the declining phase; TA, total NDVI area; TFN90, 90% of the NDVI amplitude remains; TFN50, 50% of the NDVI amplitude remains; TFN10, 10% of the NDVI amplitude remains; TFN1, 1% of the NDVI amplitude remains

Table S8: Mid-parent and best-parent heterosis of F_1 hybrids in Moinville-la-Jeulin. For each trait, are indicated the number of hybrids tested, the average heterosis, the p-value of the Student test between hybrids and parent lines, the minimal and maximal value as well as the heterosis coefficient of variation.

		Mid-parent heterosis					Best-parent heterosis				
Trait	Number of hybrids	Mean (%)	p-value	Min (%)	Max (%)	σ/μ	Mean (%)	p-value	Min (%)	Max (%)	σ/μ
YLD	81	4.2	1.2E-05	-13.0	23.6	1.9	-2.7	2.6E-03	-17.3	21.1	-2.9
GPC	81	-0.6	n.s	-10.6	11.7	-8.7	-4.1	3.5E-09	-14.2	7.5	-1.3
SW	81	0.9	4.9E-09	-2.2	4.1	1.4	-0.3	4.9E-02	-3.7	2.9	-4.5
HD	81	-2.5	1.2E-27	-6.0	0.8	-0.5	-5.3	3.4E-31	-11.4	0.0	-0.5
HT	81	8.4	2.9E-23	-3.8	21.1	0.6	0.9	n.s	-18.1	16.5	7.9
SEEDA	81	4.2	1.3E-13	-7.8	15.3	1.0	0.0	n.s	-15.5	9.6	113.1
SEEDL	81	1.2	6.1E-06	-3.8	7.6	1.8	-1.6	3.7E-06	-10.0	6.3	-1.8
SEEDW	81	2.8	1.7E-17	-5.2	9.0	0.8	1.0	7.2E-04	-6.5	6.7	2.5
TKW	81	7.0	1.2E-25	-9.1	15.3	0.6	1.8	1.2E-02	-17.7	13.7	3.5
GPY	81	3.2	9.6E-04	-13.4	23.2	2.6	-2.8	5.9E-04	-17.5	17.1	-2.5
GPSM	81	-3.2	7.5E-05	-23.2	15.7	-2.1	-8.3	2.0E-17	-23.7	9.1	-0.8
Nmax	81	2.1	3.1E-05	-6.1	13.9	2.0	-1.1	1.3E-02	-10.8	12.1	-3.5
GPA	81	1.8	7.9E-06	-8.1	14.6	1.9	-1.7	5.4E-04	-11.7	14.3	-2.5
FPA	81	-1.4	n.s	-48.6	150.4	-19.9	-18.8	7.7E-09	-60.6	117.8	-1.4
SPA	81	3.8	1.1E-07	-12.2	16.7	1.5	-4.3	3.9E-09	-16.8	7.5	-1.4
DPA	81	0.3	n.s	-18.5	35.5	24.9	-5.6	1.2E-07	-25.1	31.9	-1.5
TA	81	1.2	6.9E-03	-6.4	12.5	3.2	-2.7	1.5E-07	-11.6	7.3	-1.6
TFN90	81	7.6	2.9E-11	-13.5	33.6	1.2	-0.6	n.s	-21.6	18.0	-15.9
TFN50	81	6.1	1.3E-19	-6.6	15.3	0.7	-0.7	n.s	-12.6	9.6	-7.4
TFN10	81	5.1	3.0E-18	-4.4	14.0	0.8	-0.9	n.s	-9.5	8.6	-4.6
TFN1	81	4.4	1.7E-08	-13.0	19.5	1.4	-1.4	2.5E-02	-19.0	9.7	-3.9

YLD, grain yield; GPC, grain protein content; SW, specific weight; HD, heading date; HT, plant height; SEEDA, seed area; SEEDL, seed length; SEEDW, seed width; TKW, thousand kernel weight; GPY, grain protein yield; GPSM, grains per square meter; Nmax, maximum NDVI measured; GPA, area of the growing phase; FPA, area of the flowering phase; SPA, area of the senescence phase; DPA, area of the declining phase; TA, total NDVI area; TFN90, 90% of the NDVI amplitude remains; TFN50, 50% of the NDVI amplitude remains; TFN10, 10% of the NDVI amplitude remains; TFN1, 1% of the NDVI amplitude remains. n.s : non-significant.

Table S9: Mid-parent and Best-parent heterosis of F_1 hybrids in Arvillers. For each trait, are indicated the number of hybrids tested, the average heterosis, the p-value of the Student test between hybrids and parent lines, the minimal and maximal value as well as the heterosis coefficient of variation.

Trait	Number of hybrids	Mid-parent heterosis Mean (%)	p-value	Min (%)	Max (%)	σ/μ	Best-parent heterosis Mean (%)	p-value	Min (%)	Max (%)	σ/μ
YLD	73	6.4	2.37E-15	-9.5	16.9	0.8	2.3	2.0E-03	-15.3	14.0	2.6
GPC	67	-1.1	2.96E-03	-8.6	3.3	-2.6	-4.4	5.6E-17	-13.4	1.9	-0.7
SW	67	0.9	6.57E-10	-1.1	3.5	1.1	-0.5	5.7E-03	-2.9	2.9	-2.8
HD	73	-2.0	8.88E-26	-5.6	0.0	-0.5	-4.7	1.3E-29	-9.9	-0.7	-0.4
HT	73	6.0	4.90E-17	-15.3	16.8	0.8	-3.7	1.4E-05	-17.0	13.7	-1.8
SEEDA	67	7.5	9.27E-29	0.0	16.2	0.4	3.7	2.3E-10	-5.9	12.8	1.1
SEEDL	67	2.8	9.99E-18	-1.8	7.9	0.7	0.0	n.s	-6.5	7.0	-1216.8
SEEDW	67	4.3	9.11E-23	-1.3	10.0	0.5	2.0	3.5E-07	-5.2	7.8	1.4
TKW	67	9.4	3.39E-22	-0.4	18.7	0.5	5.4	9.0E-08	-7.1	15.8	1.1
GPY	67	6.3	2.04E-13	-11.3	17.5	0.9	2.3	1.4E-03	-11.3	15.8	2.4
GPSM	58	-2.1	7.87E-03	-13.5	9.1	-2.7	-6.8	3.0E-11	-23.7	7.0	-0.9
Nmax	69	-0.2	n.s	-4.2	3.6	-9.1	-1.1	8.2E-07	-4.8	3.2	-1.5
GPA	69	0.1	n.s	-4.7	4.2	30.1	-2.8	1.0E-11	-11.0	2.6	-1.0
FPA	69	-7.2	7.18E-10	-27.2	11.3	-1.2	-15.7	3.3E-19	-39.8	8.8	-0.7
SPA	69	5.9	1.85E-04	-20.0	35.0	2.1	-5.0	4.1E-04	-32.2	19.7	-2.2
DPA	69	-2.7	1.87E-06	-12.2	6.8	-1.6	-6.5	3.4E-16	-16.6	6.1	-0.8
TA	69	-1.9	2.25E-06	-8.9	4.6	-1.6	-4.6	4.8E-16	-12.2	4.5	-0.8
TFN90	69	5.8	6.58E-11	-9.0	21.4	1.1	1.5	n.s	-12.1	15.0	4.2
TFN50	69	4.1	2.00E-13	-2.8	14.7	0.9	-0.2	n.s	-7.5	9.5	-14.9
TFN10	69	3.1	1.33E-07	-5.6	11.3	1.4	-1.5	6.6E-03	-11.7	10.7	-2.9
TFN1	69	2.4	1.26E-03	-10.6	14.9	2.5	-2.5	9.1E-04	-16.4	12.7	-2.4

YLD, grain yield; GPC, grain protein content; SW, specific weight; HD, heading date; HT, plant height; SEEDA, seed area; SEEDL, seed length; SEEDW, seed width; TKW, thousand kernel weight; GPY, grain protein yield; GPSM, grains per square meter; Nmax, maximum NDVI measured; GPA, area of the growing phase; FPA, area of the flowering phase; SPA, area of the senescence phase; DPA, area of the declining phase; TA, total NDVI area; TFN90, 90% of the NDVI amplitude remains; TFN50, 50% of the NDVI amplitude remains; TFN10, 10% of the NDVI amplitude remains; TFN1, 1% of the NDVI amplitude remains. n.s : non-significant.

Table S10: Mid-parent and Best-parent heterosis of F_1 hybrids in Pomacle. For each trait, are indicated the number of hybrids tested, the average heterosis, the p-value of the Student test between hybrids and parent lines, the minimal and maximal value as well as the heterosis coefficient of variation.

		Mid-parent heterosis					Best-parent heterosis				
Trait	Number of hybrids	Mean (%)	p-value	Min (%)	Max (%)	σ/μ	Mean (%)	p-value	Min (%)	Max (%)	σ/μ
YLD	82	5.2	6.6E-06	-22.0	23.7	1.9	-0.8	n.s	-26.4	18.7	-11.1
GPC	79	-0.6	n.s	-6.5	8.6	-5.5	-3.5	1.4E-10	-13.5	8.4	-1.2
SW	79	0.5	3.6E-07	-1.8	3.1	1.6	-0.4	1.2E-03	-3.2	1.7	-2.6
HD	82	-2.6	1.8E-28	-5.8	1.1	-0.5	-5.6	3.2E-34	-9.7	0.0	-0.4
HT	82	6.5	1.3E-17	-6.7	21.1	0.8	-1.7	4.2E-02	-17.0	18.3	-4.4
SEEDA	78	5.7	1.4E-16	-5.8	23.7	0.8	0.3	n.s	-13.5	16.5	16.0
SEEDL	78	2.3	1.4E-17	-2.8	7.3	0.8	-1.0	3.9E-03	-9.0	3.2	-2.9
SEEDW	78	3.1	6.1E-15	-4.3	14.5	0.9	1.0	9.1E-03	-7.1	12.6	3.3
TKW	79	7.6	6.2E-27	-2.2	15.1	0.5	2.4	6.9E-05	-11.4	12.2	2.3
GPY	79	4.8	3.7E-05	-20.0	24.2	2.0	-0.3	n.s	-21.7	21.3	-34.7
GPSM	79	-2.4	1.2E-02	-24.3	14.9	-3.4	-7.2	1.8E-12	-27.1	7.9	-1.1
Nmax	80	2.8	4.8E-06	-12.9	21.9	1.8	0.1	n.s	-13.0	13.5	31.3
GPA	80	2.5	3.7E-06	-13.4	17.1	1.8	-1.0	1.9E-02	-15.4	11.4	-3.7
FPA	80	-0.8	n.s	-21.8	22.1	-9.3	-7.4	2.8E-12	-29.3	15.1	-1.1
SPA	80	0.5	n.s	-24.5	24.5	20.4	-4.2	5.1E-04	-25.2	15.9	-2.5
DPA	80	-0.4	n.s	-15.6	10.1	-13.0	-5.0	1.1E-11	-25.3	9.0	-1.1
TA	80	1.1	1.1E-02	-11.5	12.3	3.4	-2.3	3.1E-07	-16.4	5.7	-1.6
TFN90	80	4.2	1.3E-06	-14.8	21.8	1.7	-1.7	5.3E-02	-21.0	14.5	-4.5
TFN50	80	3.1	1.7E-07	-6.9	13.7	1.5	-1.3	3.3E-02	-13.3	11.2	-4.1
TFN10	80	2.5	5.0E-05	-8.5	19.2	2.1	-1.5	2.1E-02	-12.6	16.9	-3.8
TFN1	80	2.0	5.6E-03	-11.6	23.3	3.1	-1.8	2.2E-02	-14.0	21.3	-3.8

YLD, grain yield; GPC, grain protein content; SW, specific weight; HD, heading date; HT, plant height; SEEDA, seed area; SEEDL, seed length; SEEDW, seed width; TKW, thousand kernel weight; GPY, grain protein yield; GPSM, grains per square meter; Nmax, maximum NDVI measured; GPA, area of the growing phase; FPA, area of the flowering phase; SPA, area of the senescence phase; DPA, area of the declining phase; TA, total NDVI area; TFN90, 90% of the NDVI amplitude remains; TFN50, 50% of the NDVI amplitude remains; TFN10, 10% of the NDVI amplitude remains; TFN1, 1% of the NDVI amplitude remains. n.s : non-significant.

Table S11: Mid-parent and Best-parent heterosis of the five best F_1 hybrids for yield in the three locations.

Trait	Mid-parent heterosis	Best-parent heterosis
YLD	10.5	6.9
GPC	-1.0	-3.3
SW	1.1	-0.2
HD	-1.6	-5.7
HT	7.4	-1.9
SEEDA	5.4	2.4
SEEDL	1.2	-0.4
SEEDW	3.6	2.5
TKW	9.4	5.5
GPY	7.0	5.1
GPSM	-1.0	-4.6
Nmax	1.6	0.1
GPA	1.1	-2.1
FPA	-1.8	-13.1
SPA	5.1	-2.9
DPA	1.3	-3.8
TA	1.3	-1.7
TFN90	6.8	1.2
TFN50	6.5	-0.5
TFN10	6.2	-1.6
TFN1	6.1	-2.4

YLD, grain yield; GPC, grain protein content; SW, specific weight; HD, heading date; HT, plant height; SEEDA, seed area; SEEDL, seed length; SEEDW, seed width; TKW, thousand kernel weight; GPY, grain protein yield; GPSM, grains per square meter; Nmax, maximum NDVI measured; GPA, area of the growing phase; FPA, area of the flowering phase; SPA, area of the senescence phase; DPA, area of the declining phase; TA, total NDVI area; TFN90, 90% of the NDVI amplitude remains; TFN50, 50% of the NDVI amplitude remains; TFN10, 10% of the NDVI amplitude remains; TFN1, 1% of the NDVI amplitude remains. n.s : non-significant.
