# Supplementary figures and images for: Dissecting Bread Wheat Heterosis through the Integration of Agronomic and Physiological Traits
Source: Biology (Basel). 2021 Sep 13;10(9):907. doi: 10.3390/biology10090907 (PMC8465846; doi:10.3390/biology10090907)

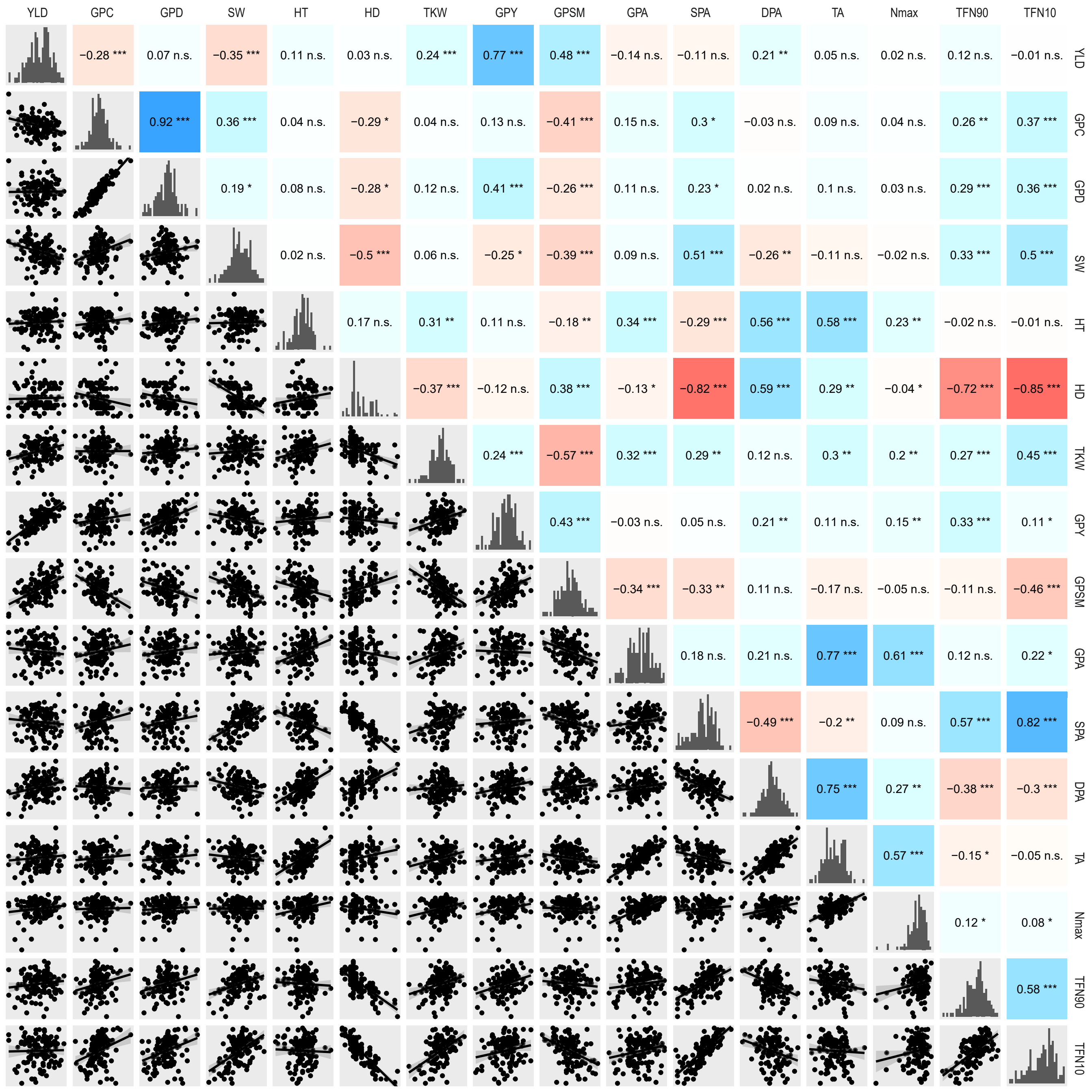

Supplement: Supplementary file 1 [file biology-10-00907-s001.zip › supplemental data/Figure S1.pdf]

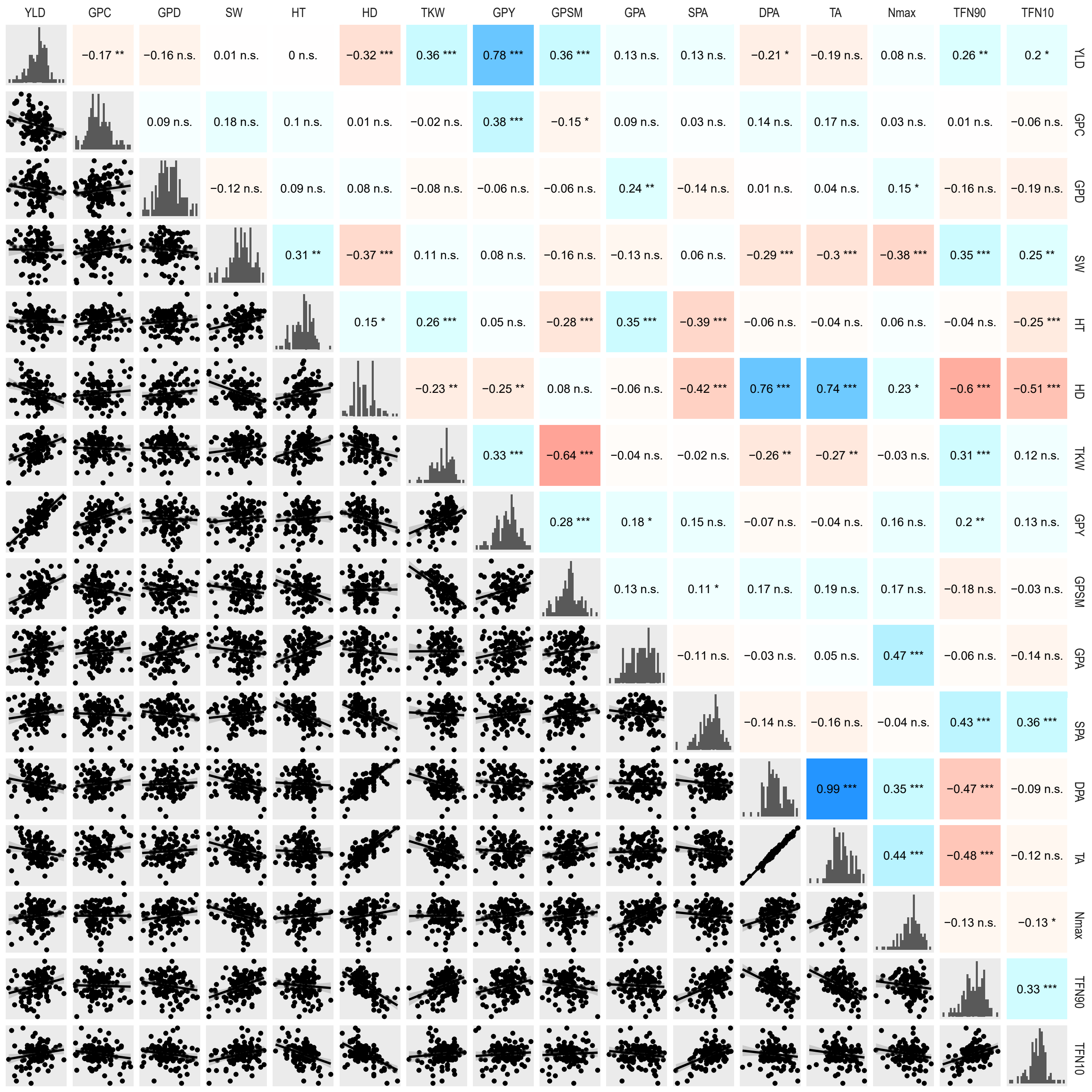

Supplement: Supplementary file 1 [file biology-10-00907-s001.zip › supplemental data/Figure S2.pdf]

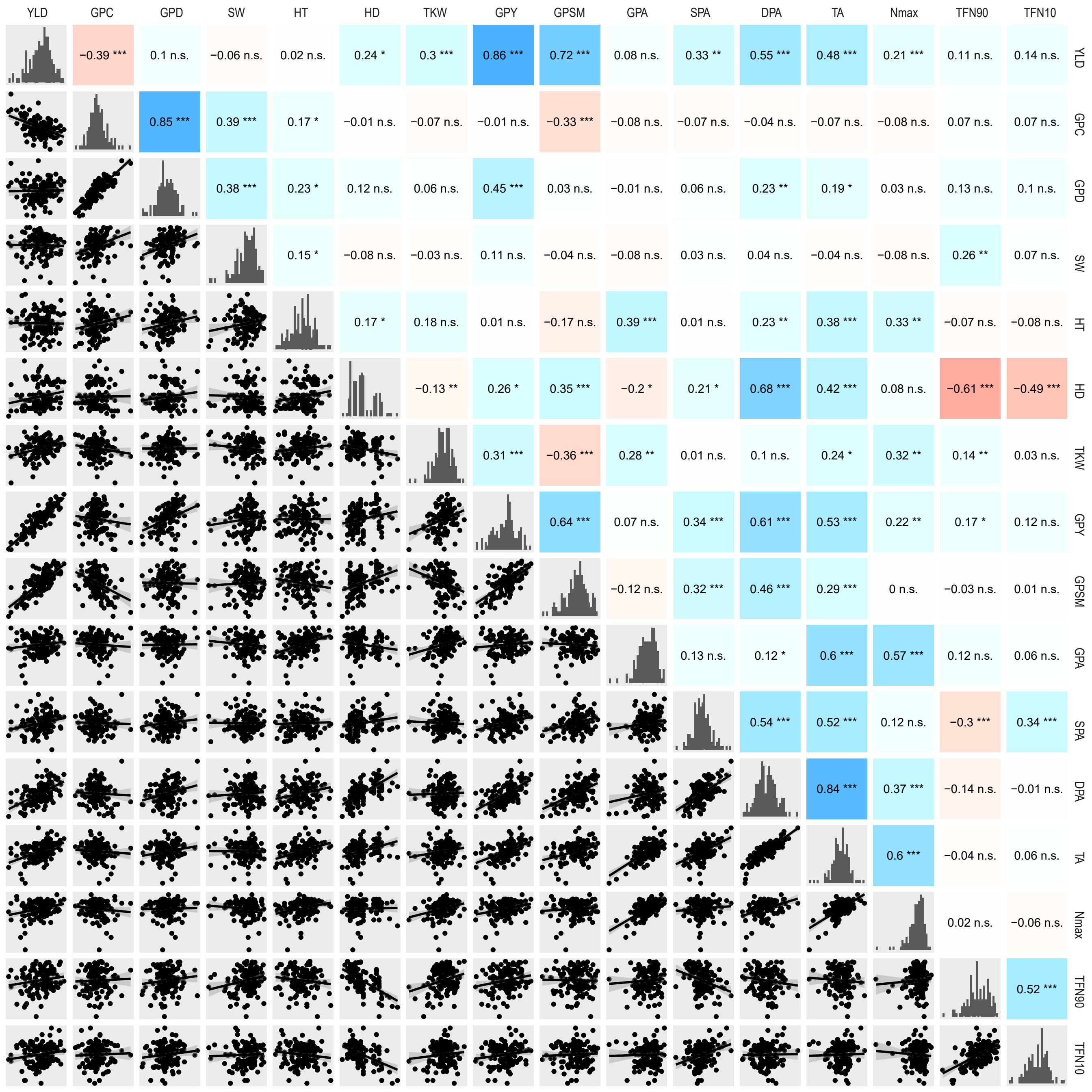

Supplement: Supplementary file 1 [file biology-10-00907-s001.zip › supplemental data/Figure S3.pdf]

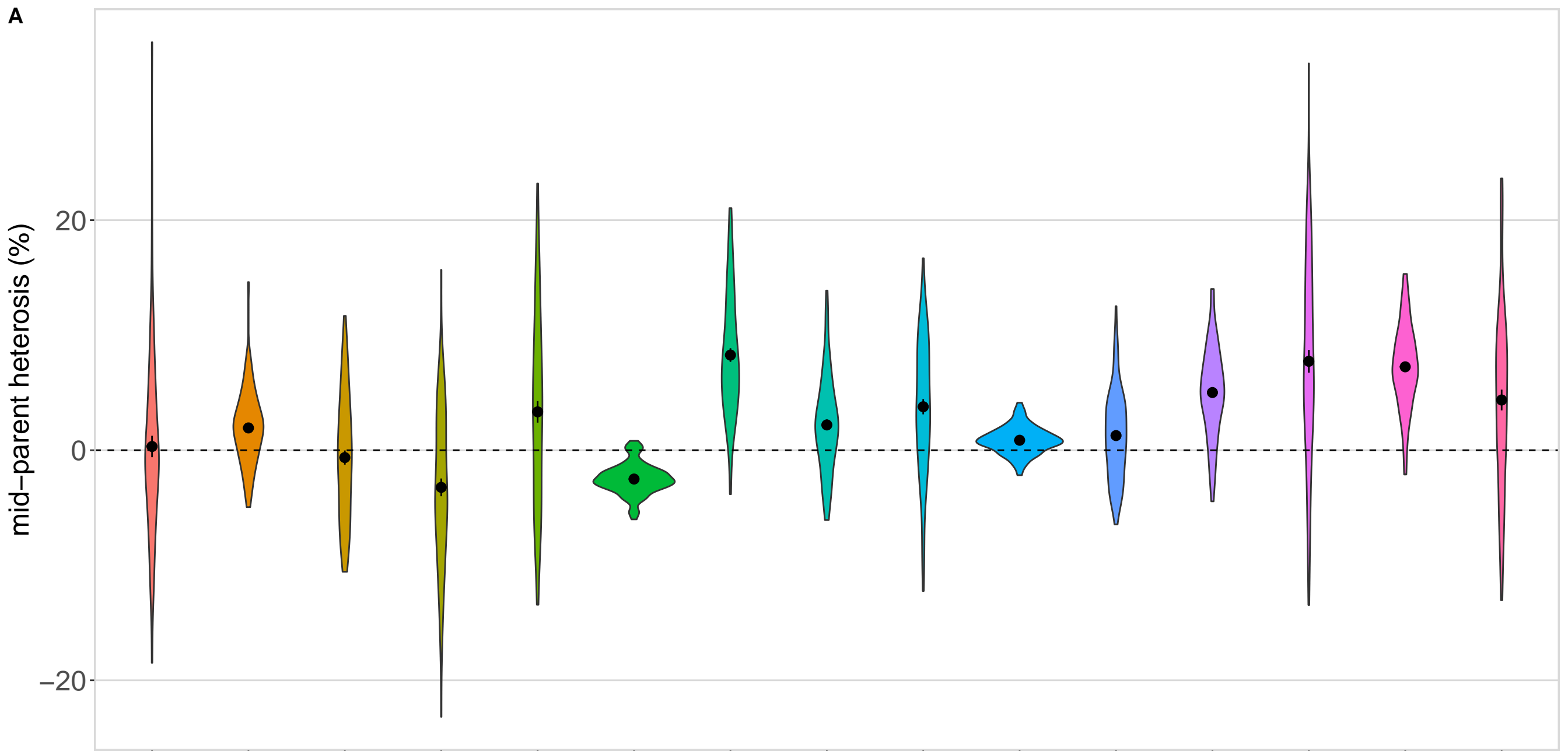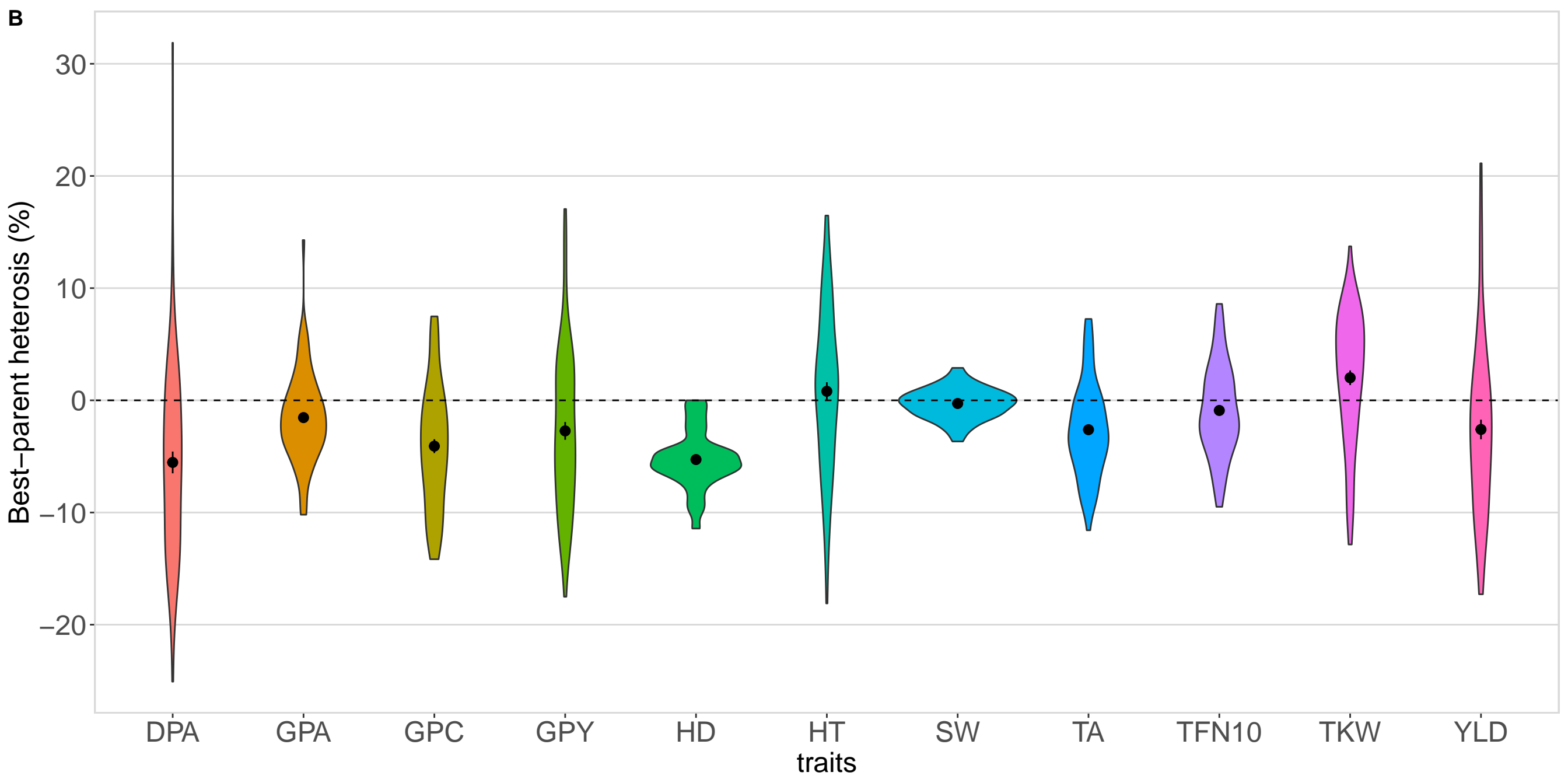

Supplement: Supplementary file 1 [file biology-10-00907-s001.zip › supplemental data/Figure S4.pdf]

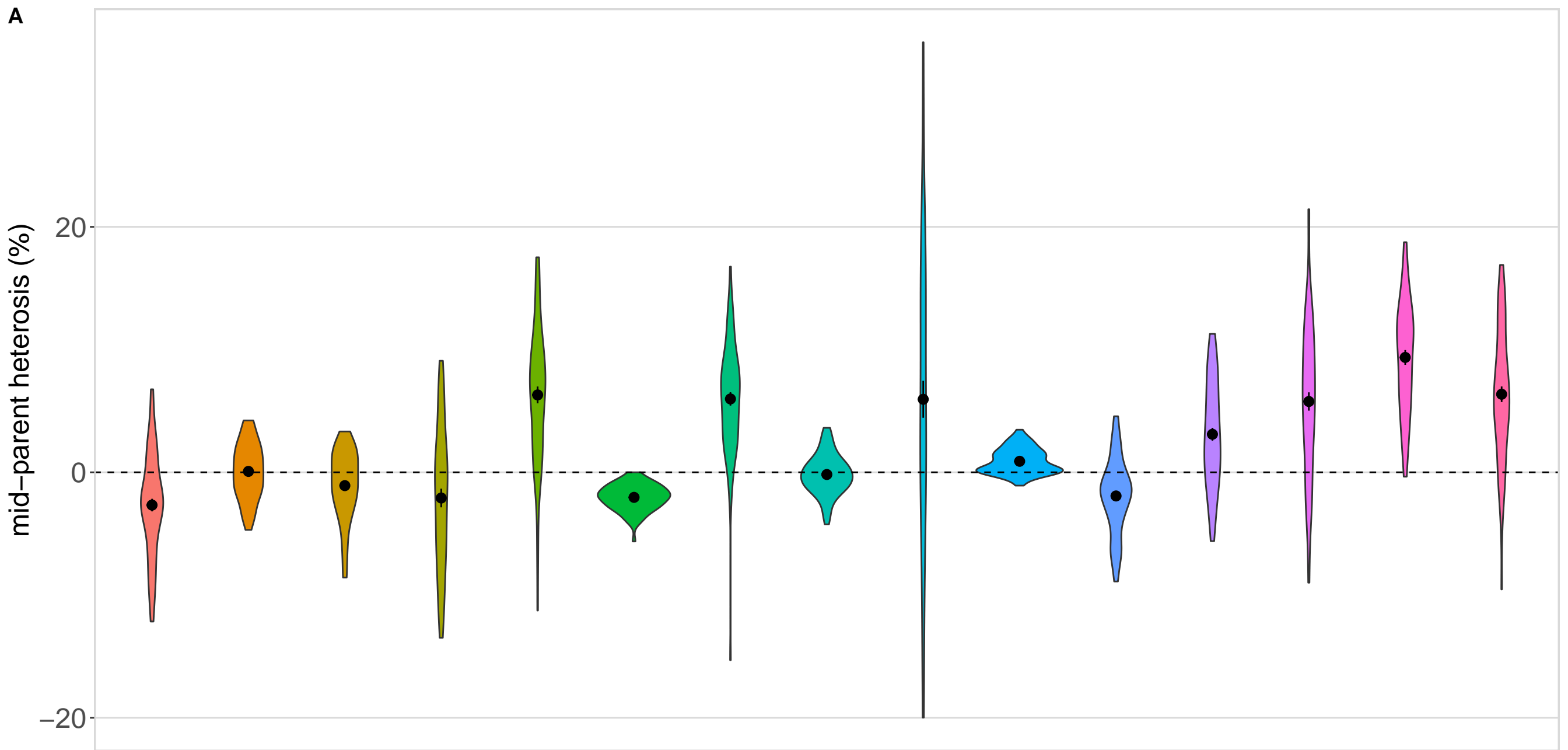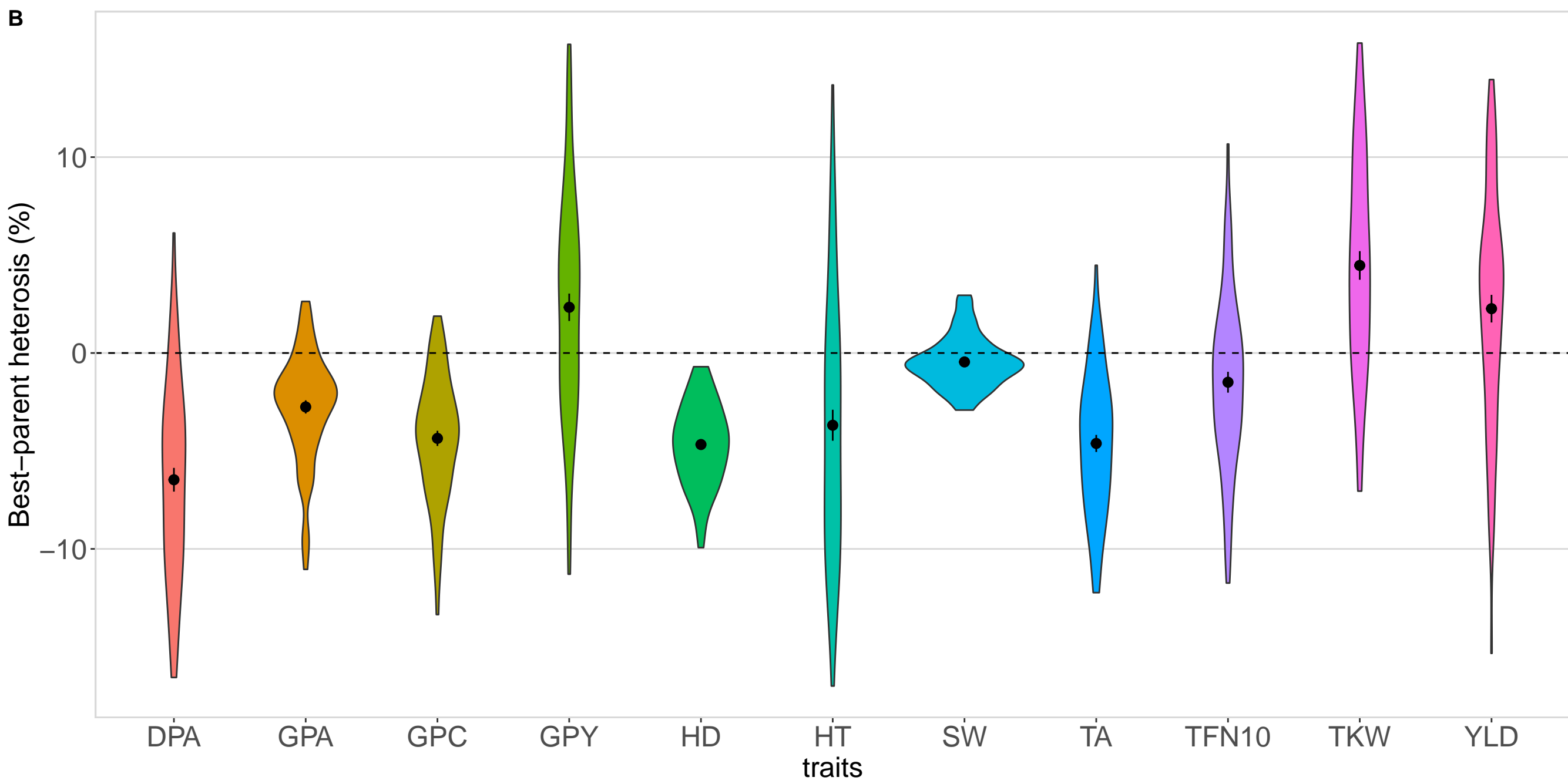

Supplement: Supplementary file 1 [file biology-10-00907-s001.zip › supplemental data/Figure S5.pdf]

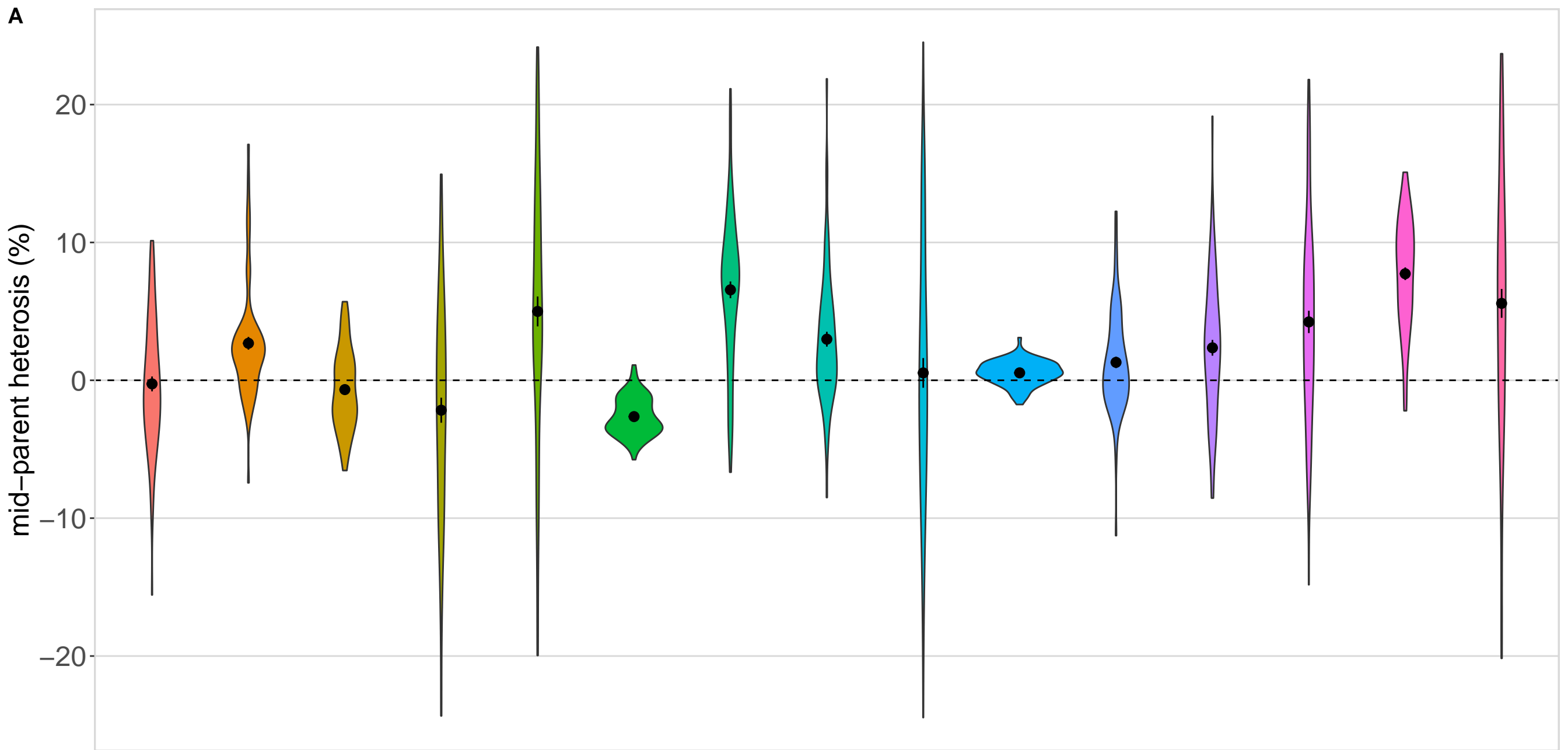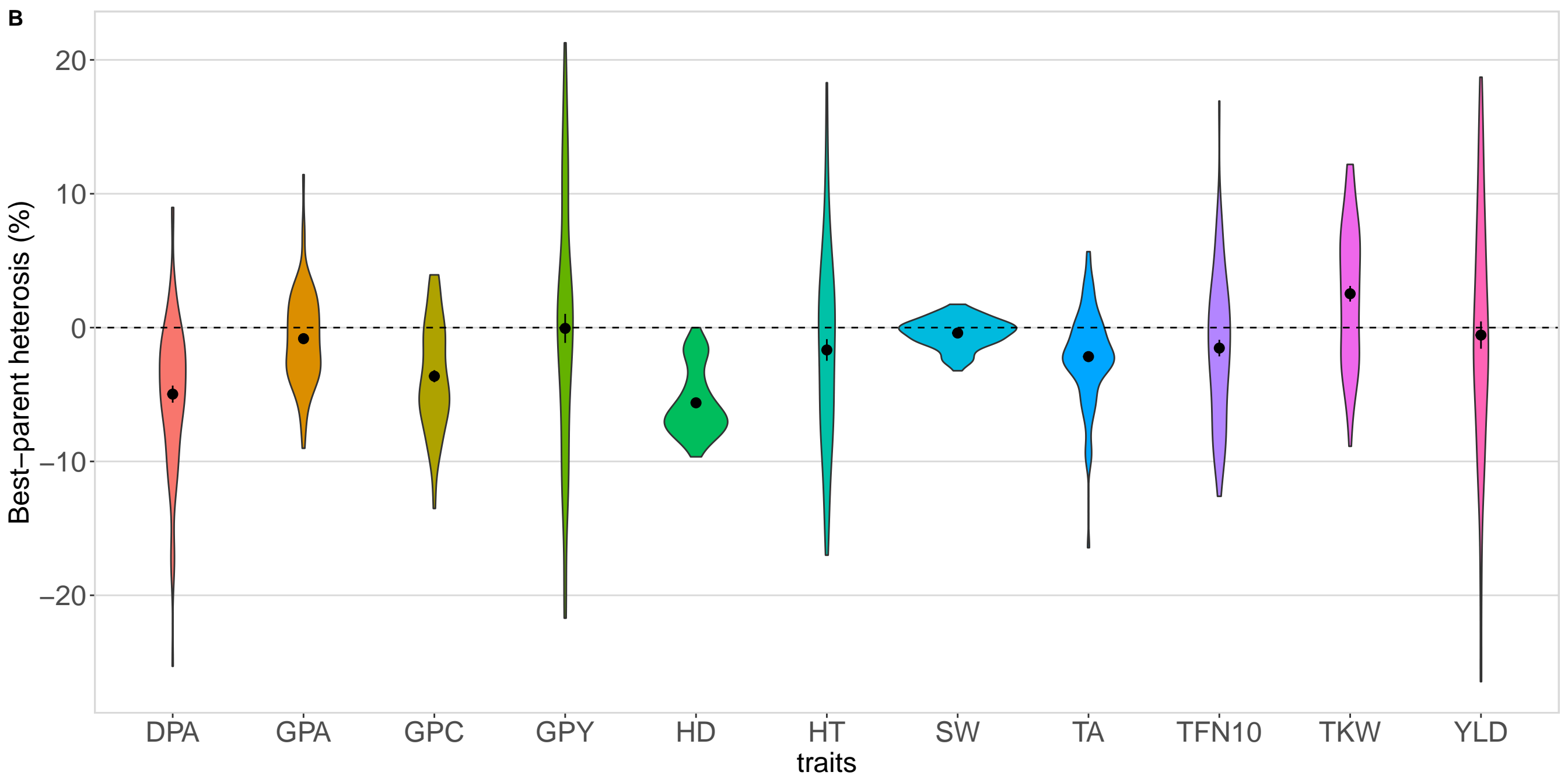

Supplement: Supplementary file 1 [file biology-10-00907-s001.zip › supplemental data/Figure S6.pdf]

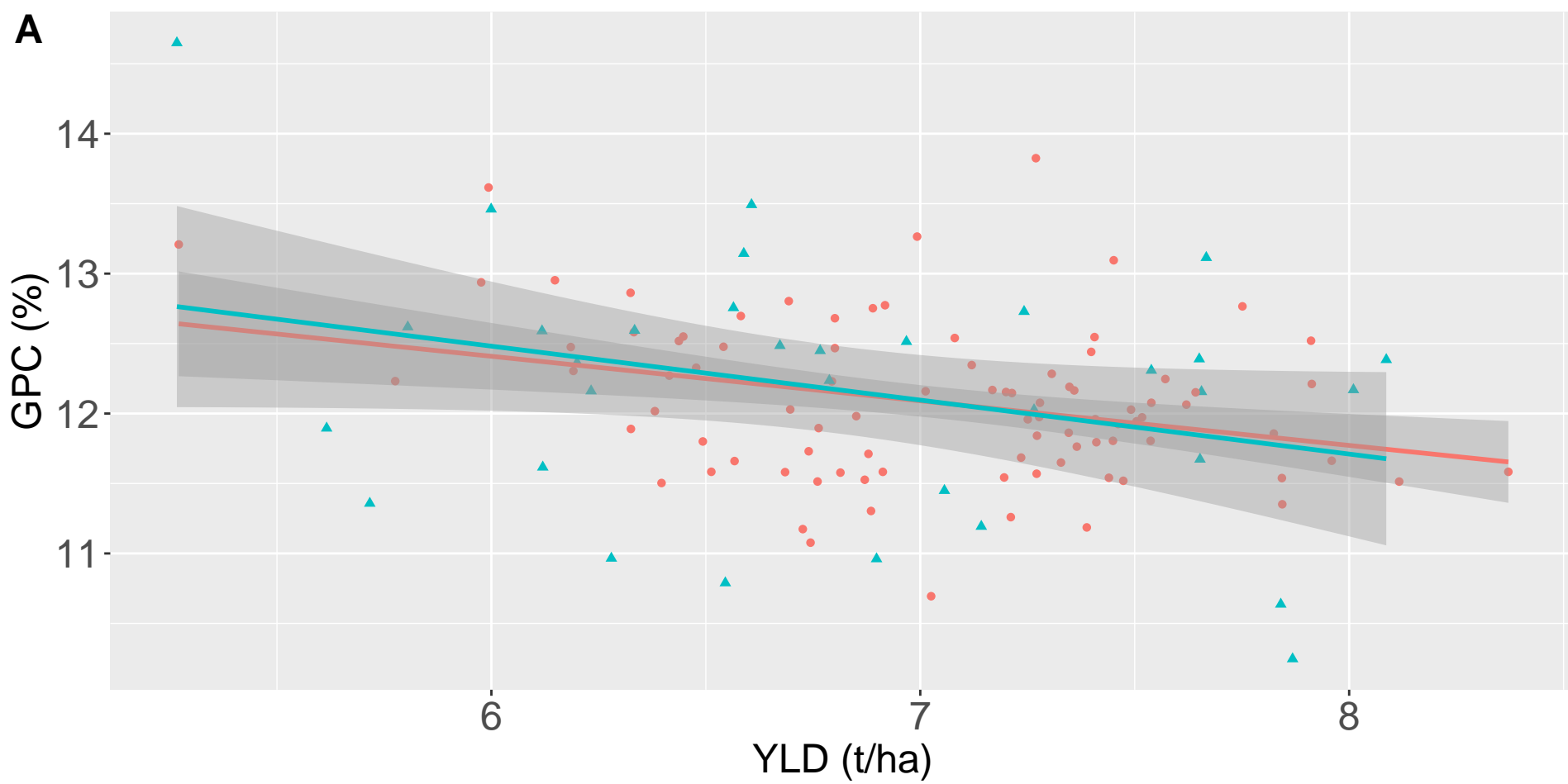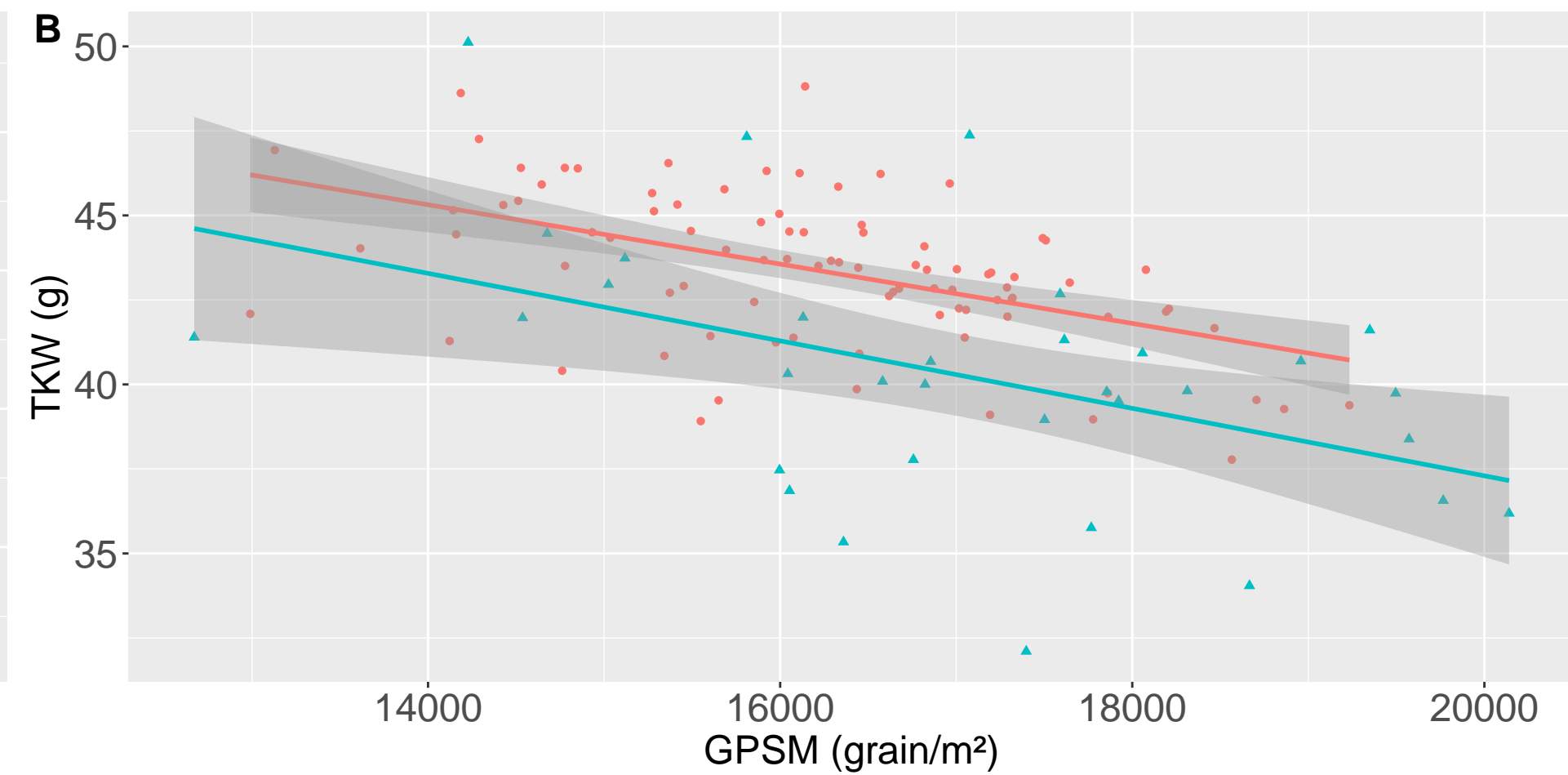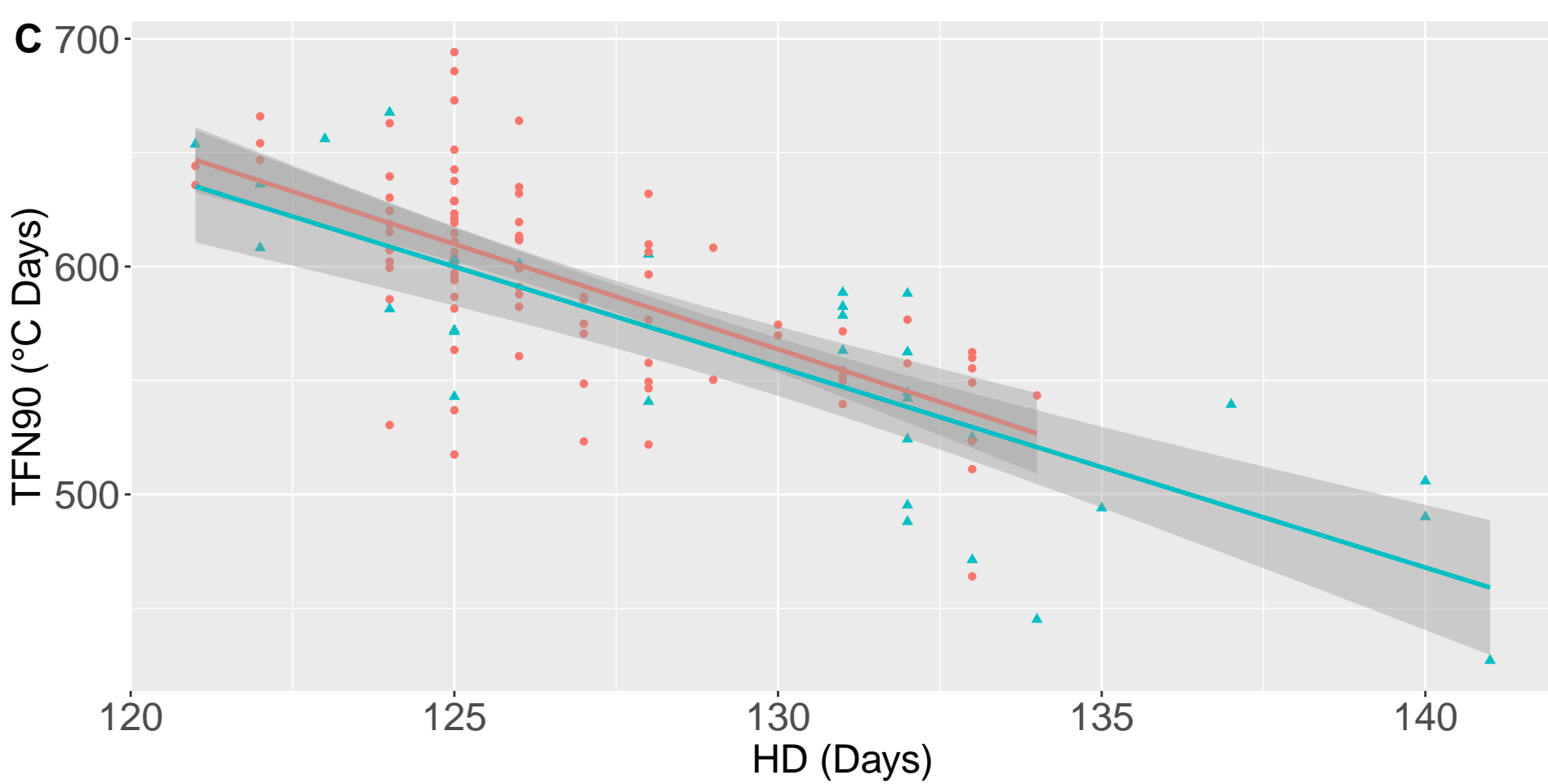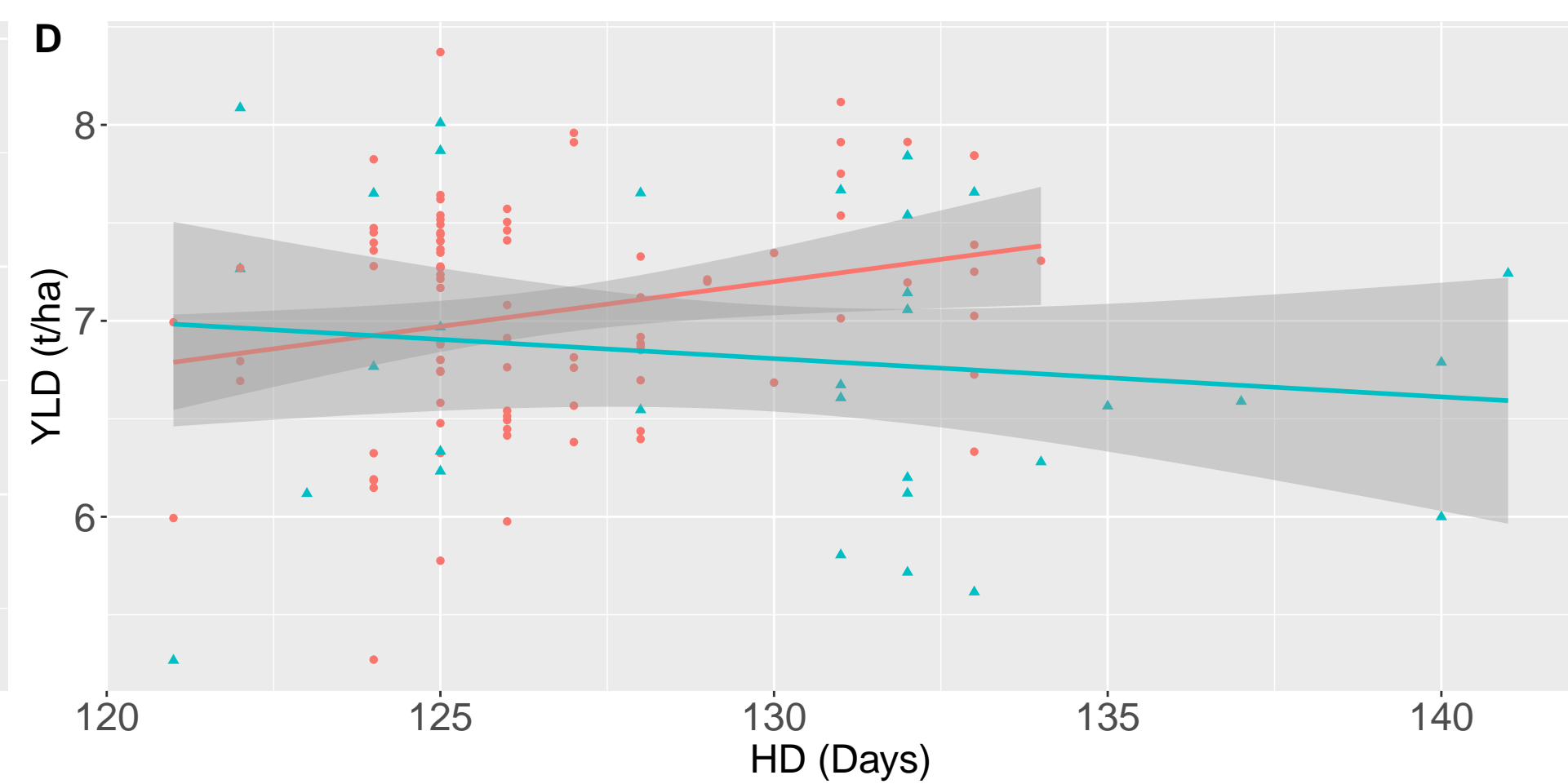

Supplement: Supplementary file 1 [file biology-10-00907-s001.zip › supplemental data/Figure S7.pdf]

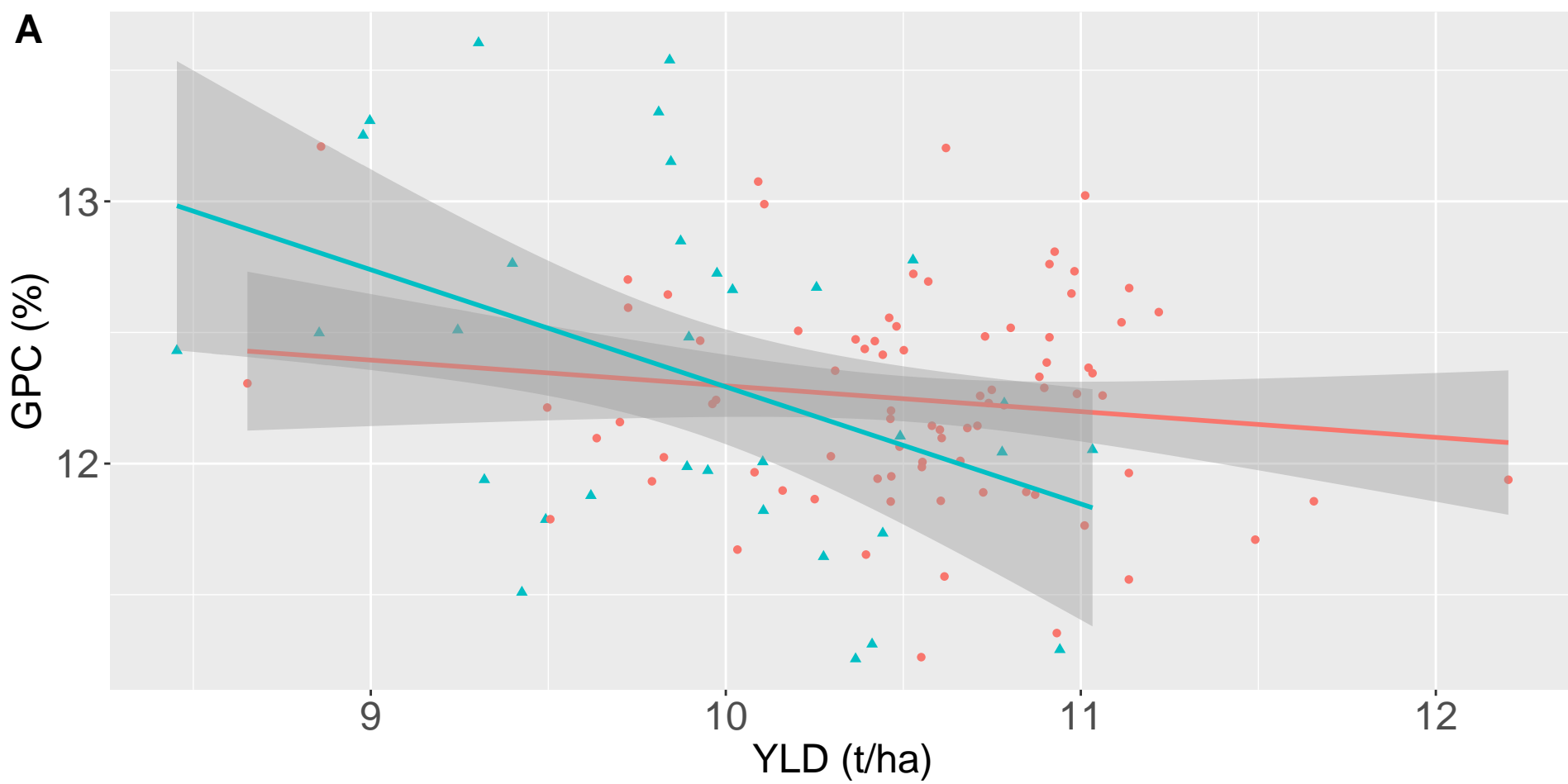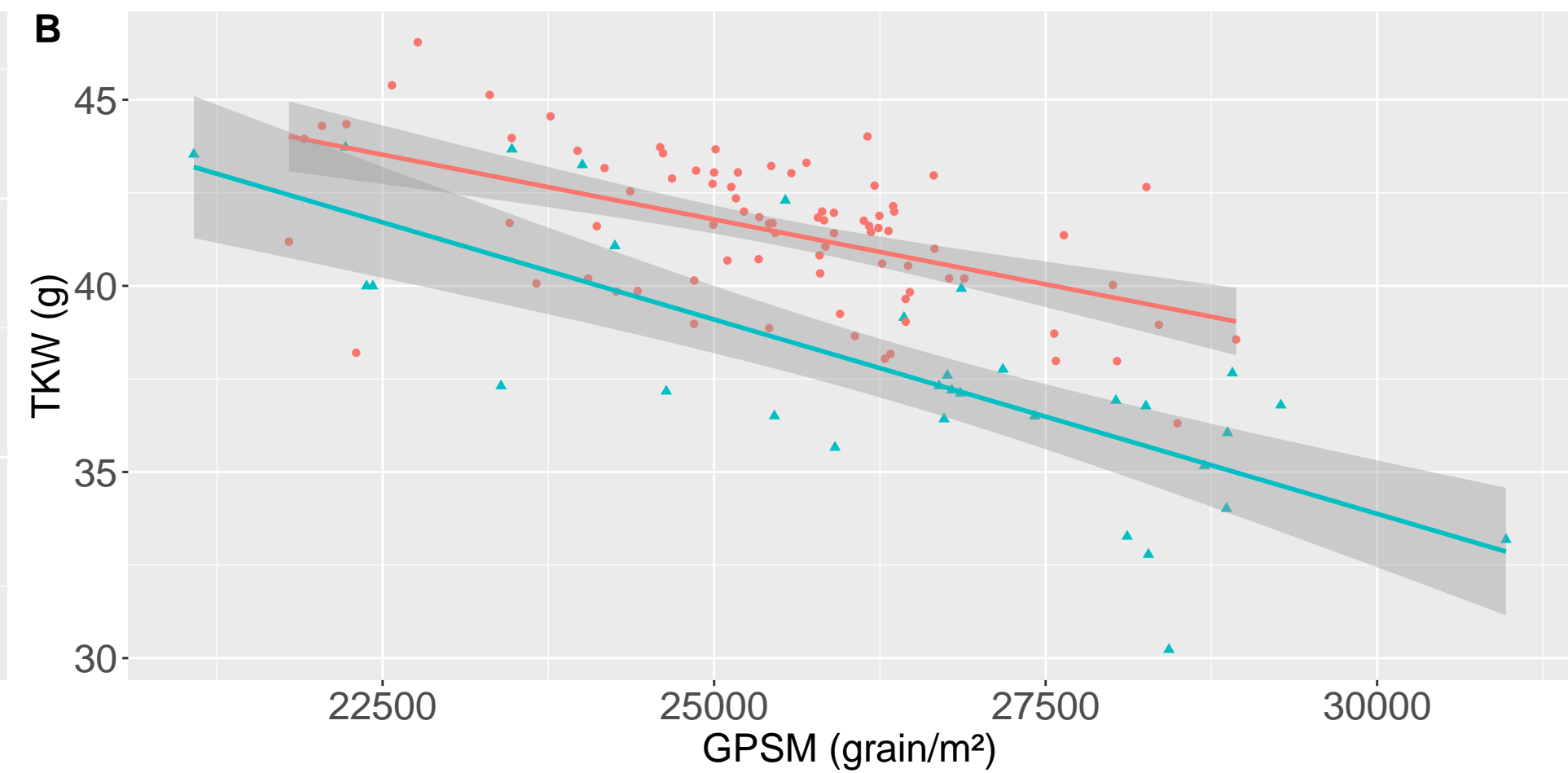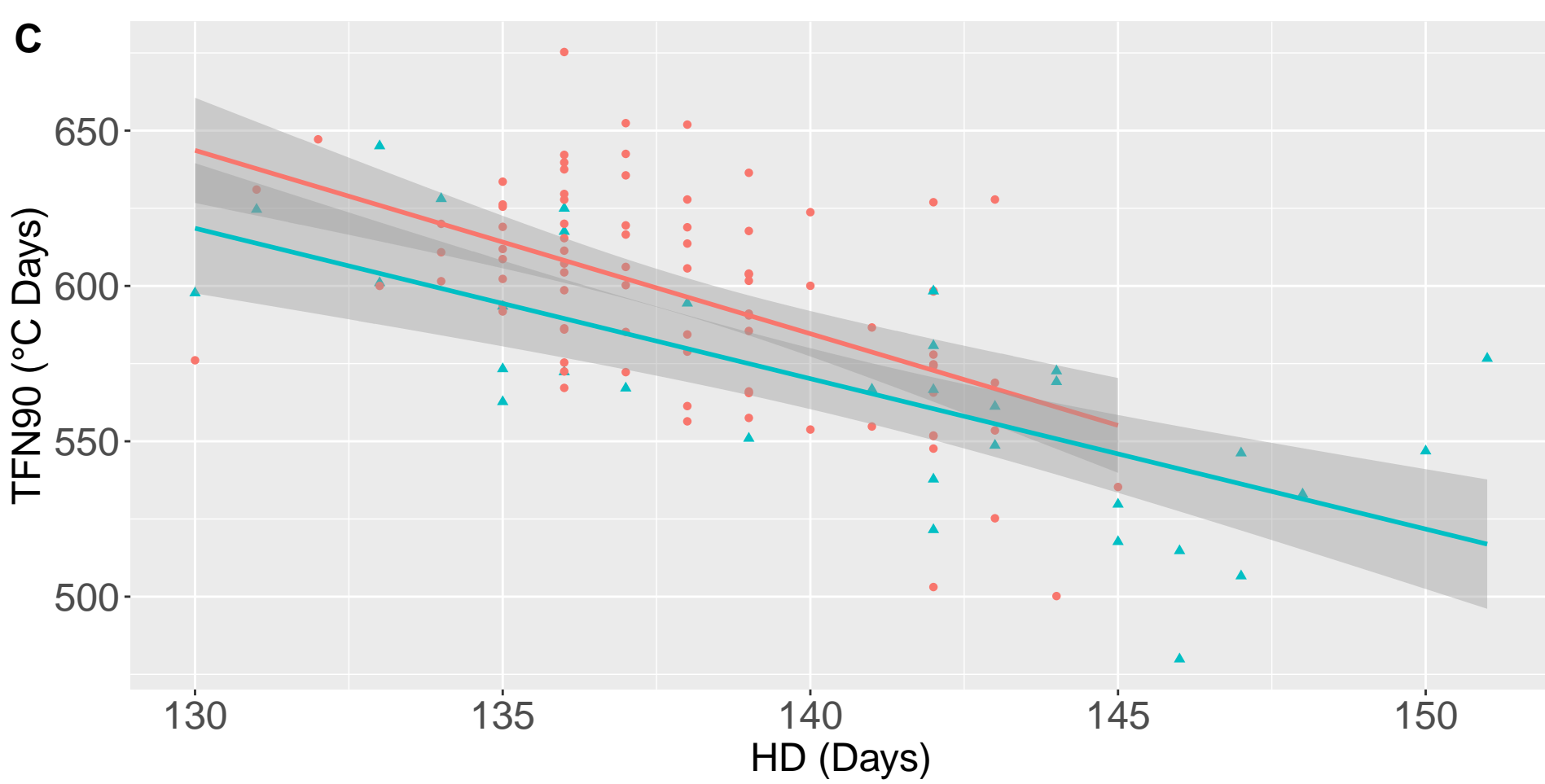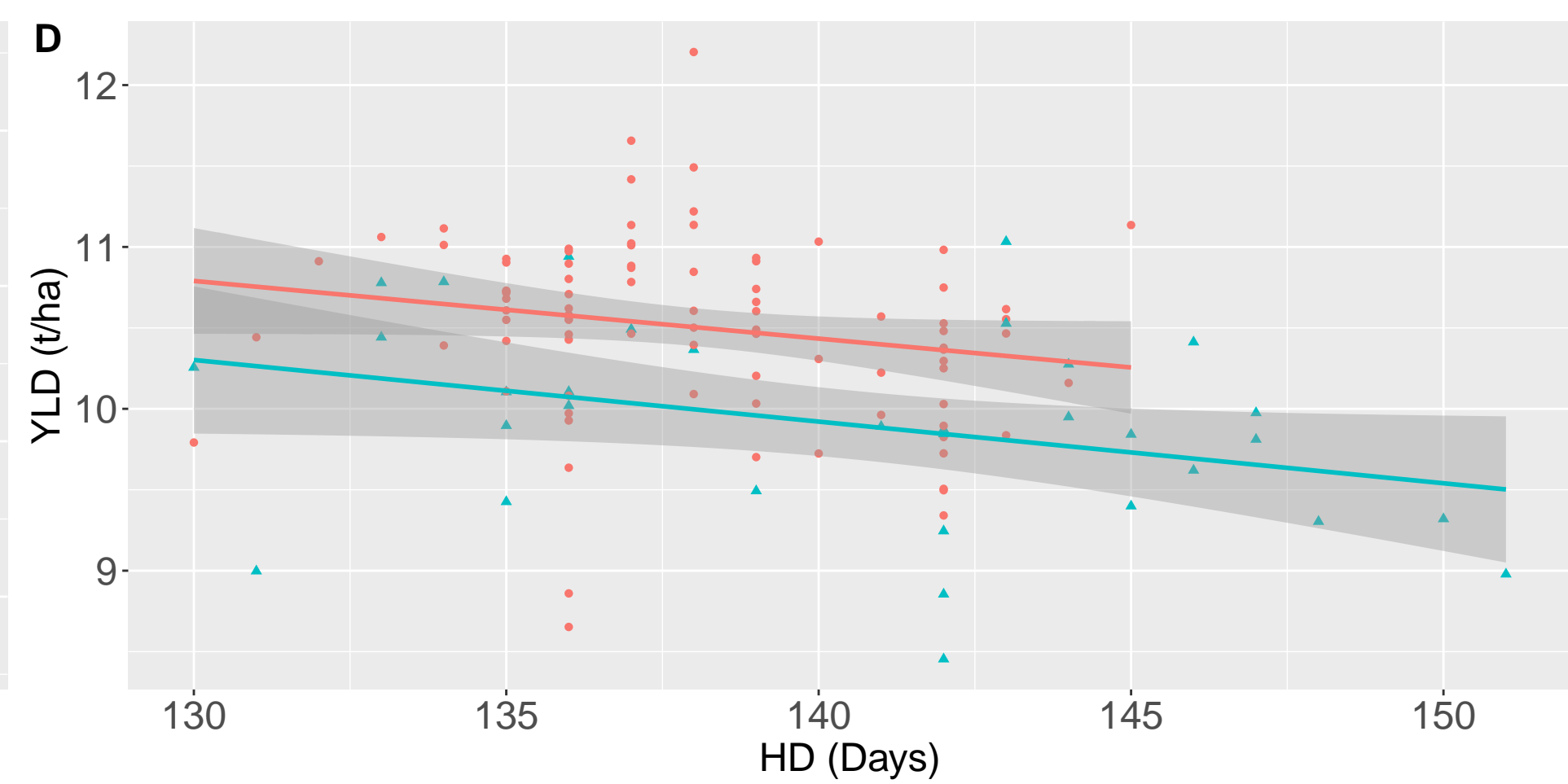

Supplement: Supplementary file 1 [file biology-10-00907-s001.zip › supplemental data/Figure S8.pdf]

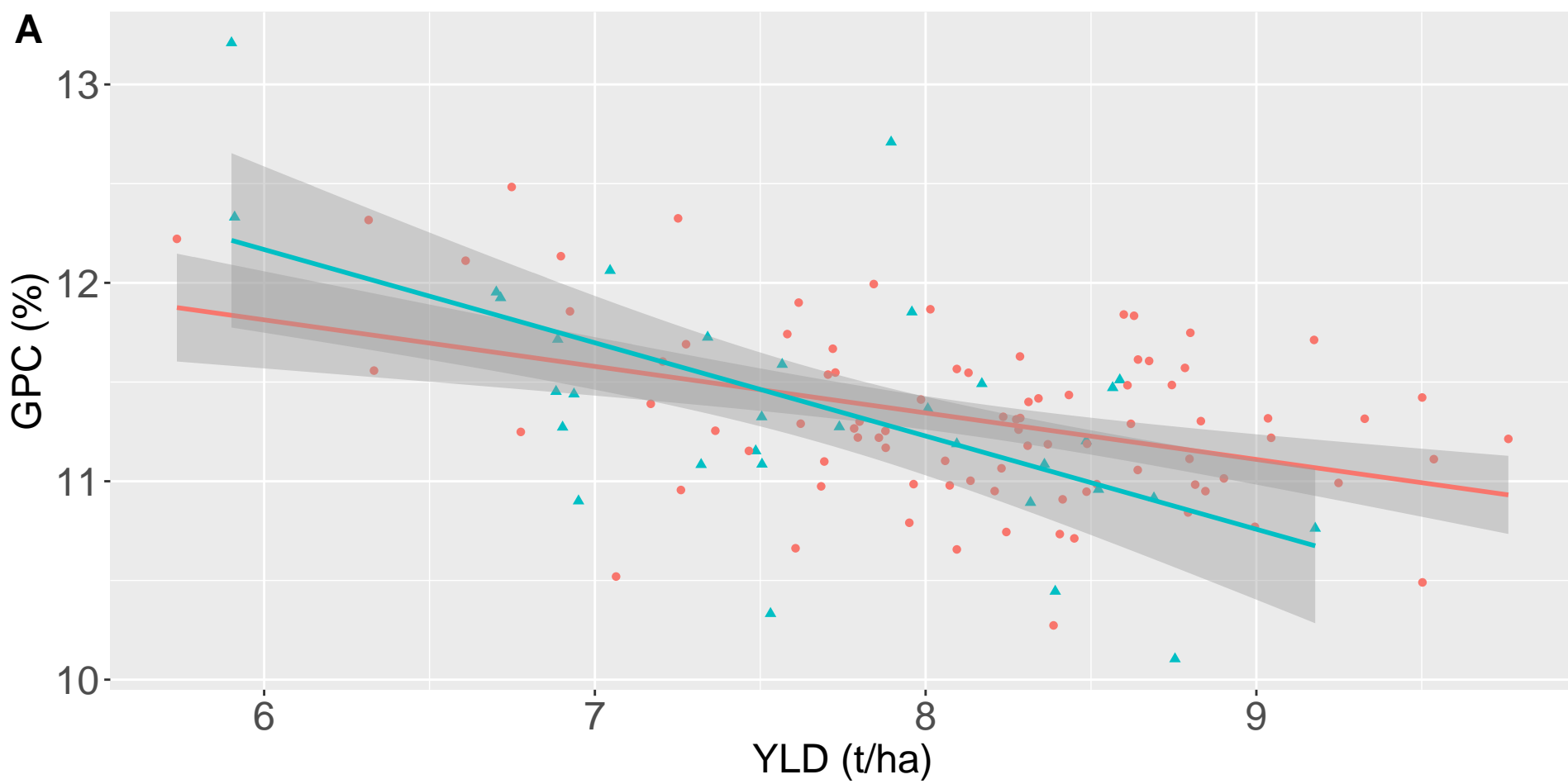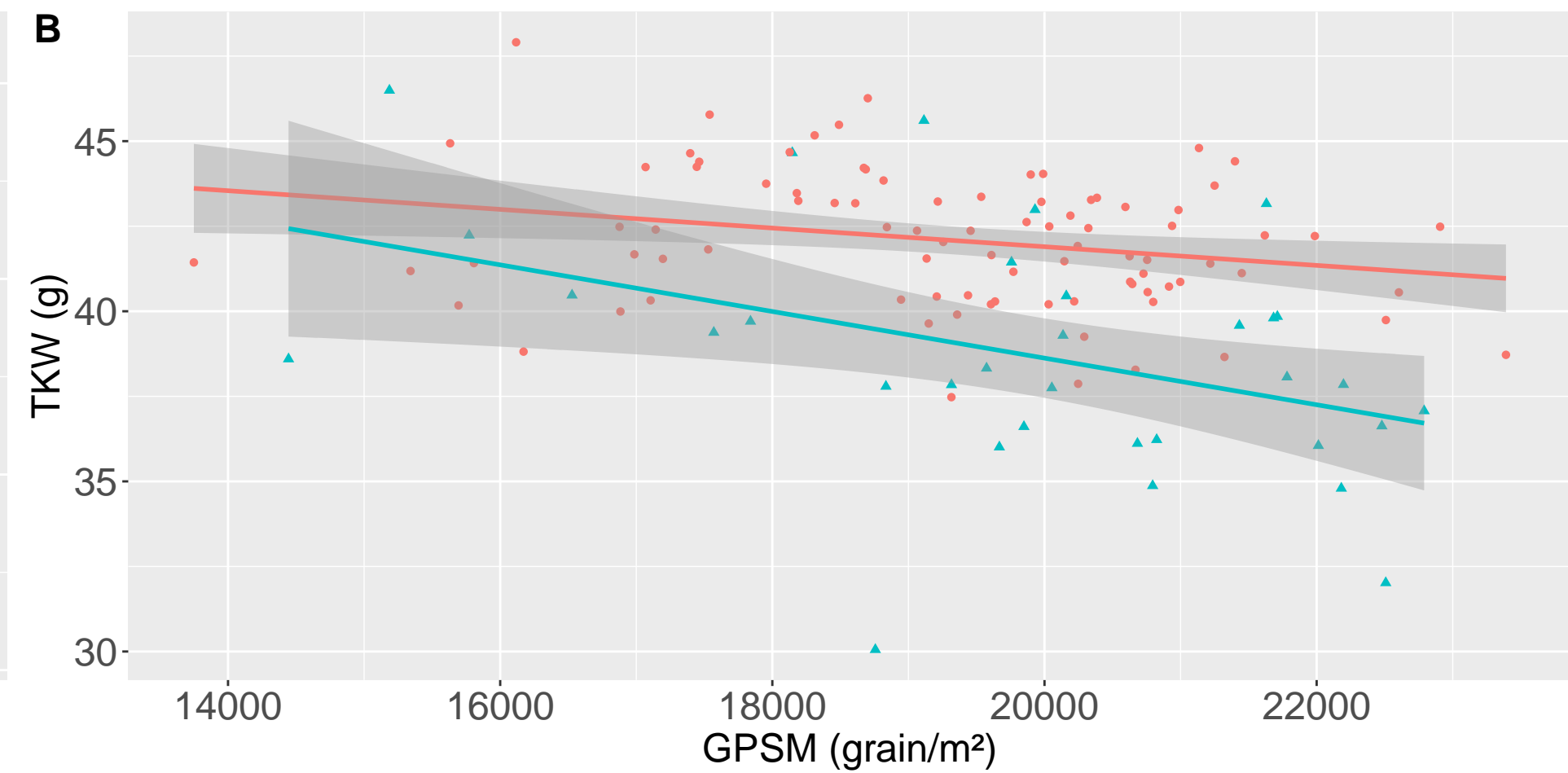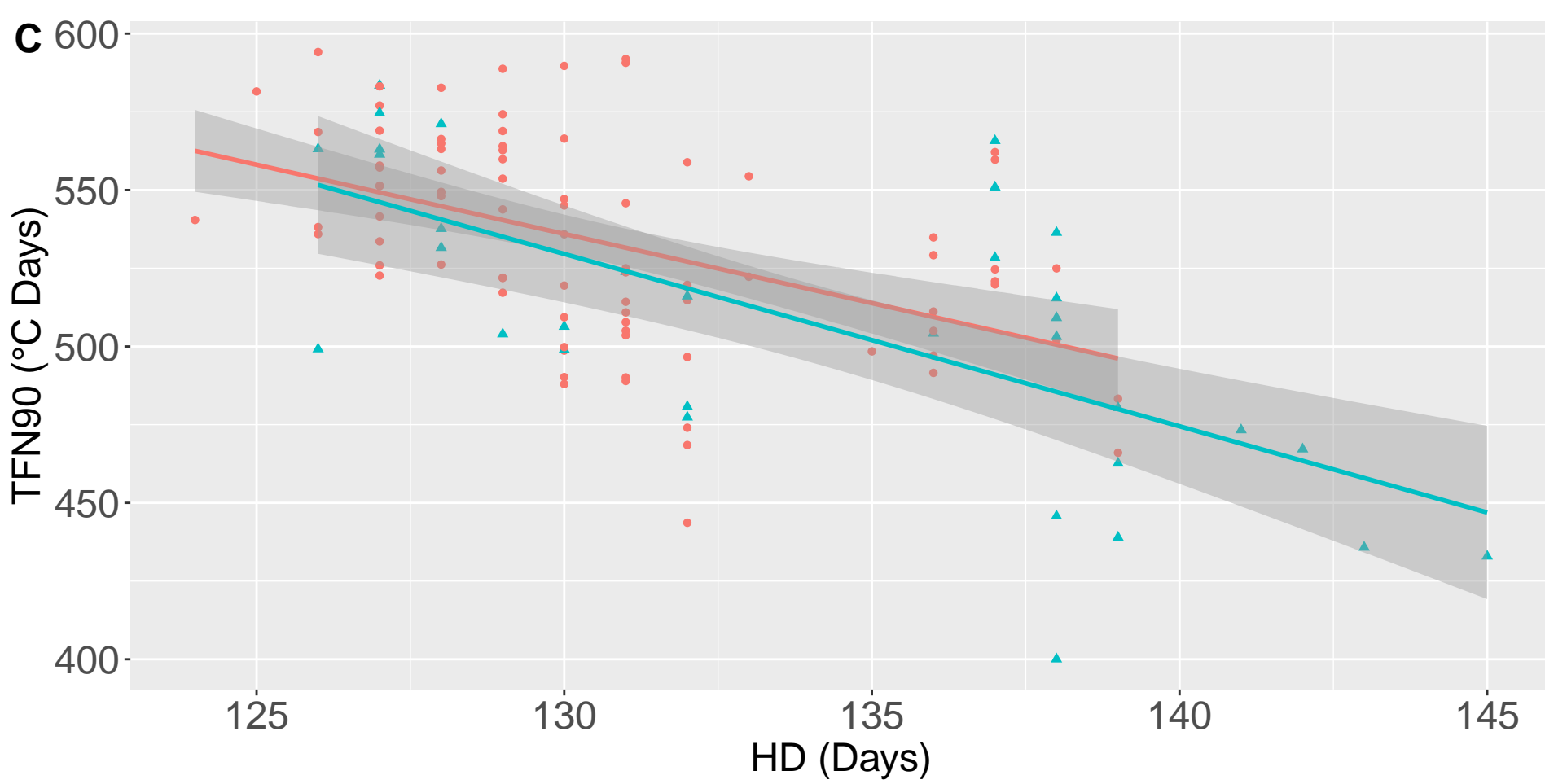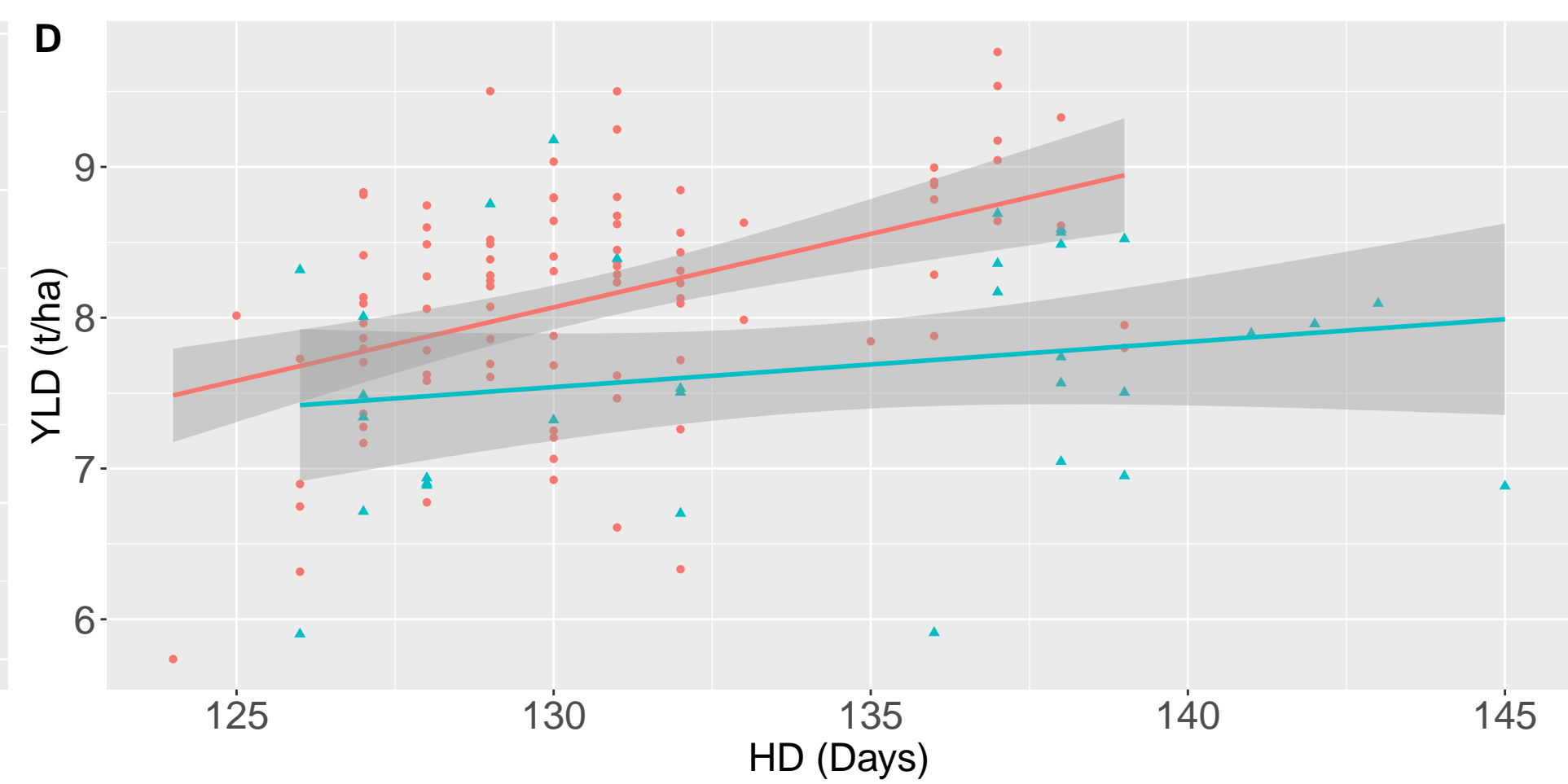

Supplement: Supplementary file 1 [file biology-10-00907-s001.zip › supplemental data/Figure S9.pdf]
